# Molecular Mechanisms and Molecular Subtype-Specific Responses to Paclitaxel in Breast Cancer Cells

**DOI:** 10.3390/molecules31142431

**Published:** 2026-07-11

**Authors:** Kezban Uçar Çifçi, Ayşe Büşranur Çelik, Levent Gülüm, Saniye Koç Ada, Mihrican Demir, Yusuf Tutar

**Affiliations:** 1 Division of Basic Sciences and Health, Hemp Research Institute, Yozgat Bozok University, Yozgat 66900, Türkiye; kezban.u.cifci@bozok.edu.tr; 2 Division of Molecular Medicine, Institute of Health Sciences, University of Health Sciences, Istanbul 34668, Türkiye; 3 Division of Molecular Biology and Genetics, Faculty of Hamidiye, Institute of Health Sciences, University of Health Sciences, Istanbul 34668, Türkiye; aysebusranur_celik25@erdogan.edu.tr; 4 Department of Basic Medical Sciences, Division of Biochemistry, Faculty of Medicine, Recep Tayyip Erdogan University, Rize 53020, Türkiye; 5 Department of Crop and Animal Production, Mudurnu Süreyya Astarcı Vocational School, Bolu Abant Izzet Baysal University, Bolu 14030, Türkiye; leventgulum@ibu.edu.tr; 6 Department of Basic Medical Sciences, Division of Biochemistry, Faculty of Medicine, İstanbul Medeniyet University, Istanbul 34700, Türkiye; saniye.ada@medeniyet.edu.tr; 7 Molecular Medicine Program, Health Sciences Institutes, İstanbul Medeniyet University, Istanbul 34700, Türkiye; mihricandemir5@gmail.com; 8 Molecular Medicine Program, Health Sciences Institutes, Recep Tayyip Erdogan University, Rize 53100, Türkiye; 9 Recep Tayyip Erdogan University Training and Research Hospital, Rize 53100, Türkiye; 10 Molecular Oncology Program, Health Sciences Institutes, Recep Tayyip Erdogan University, Rize 53100, Türkiye

**Keywords:** PTX, apoptosis, breast cancer, energy metabolism, cell cycle, drug resistance, United Nations Sustainable Development Goal 3 (UN SDG 3), SDG Target 3.4

## Abstract

Paclitaxel (PTX), a taxane-derived chemotherapeutic agent, is frequently used in the treatment of breast cancer (BC). Its anticancer effects are primarily associated with microtubule stabilization, disruption of cell-cycle progression, and triggering of apoptotic cell death. In the present study, we investigated the effects of PTX on the expression of genes involved in cancer-related pathways, energy metabolism, and drug resistance in four molecularly distinct BC cell lines: MCF-7, BT-474, SK-BR-3, and MDA-MB-231. The half-maximal inhibitory concentrations (IC_50_) of PTX in BC cell lines and the non-tumorigenic hTERT-HME1 breast epithelial cell line were determined by the MTT assay to assess cell cytotoxicity. BC cells were exposed to nine different concentrations of PTX for 24, 48, and 72 h to evaluate concentration- and time-dependent effects. Following treatment, total RNA was isolated and converted into cDNA, and RT-qPCR analysis was performed to investigate PTX-mediated alterations in the expression of genes associated with cancer-related pathways. The impact of PTX on the cell-cycle phase distribution and apoptotic cell death was evaluated by flow cytometry. Treatment with PTX for 48 h at concentrations of 12.60 nM in MCF-7, 5.09 nM in BT-474, 16.09 nM in SK-BR-3, and 36.66 nM in MDA-MB-231 cells reduced cell viability and increased apoptosis. PTX treatment also altered the expression of genes involved in apoptosis, cell-cycle regulation, angiogenesis, epithelial–mesenchymal transition, hypoxia-related signaling, energy metabolism, telomere maintenance, and therapy resistance. Collectively, these findings demonstrate that PTX elicits heterogeneous molecular and cellular responses across molecularly distinct BC cell lines, particularly in cell viability, apoptosis, metabolic regulation, and treatment response. These in vitro findings suggest potential molecular mechanisms that could explain why some cells are more sensitive to PTX than others, but further experimental and clinical validation is needed to confirm this.

## 1. Introduction

Breast cancer (BC) is the most frequently diagnosed malignancy among women and remains a leading cause of cancer-related mortality. Its biological heterogeneity is reflected in distinct gene-expression and receptor profiles. BC is commonly classified into luminal A, luminal B, HER2-enriched, and basal-like/triple-negative subtypes. Luminal B tumors may be either HER2-negative or HER2-positive, depending on their hormone-receptor expression and proliferative characteristics [1]. Most BCs are ER+, and their growth largely depends on estrogen signaling [2]. Treatment responses vary considerably among BC molecular subtypes.

For the treatment of HR+ (ER+ and/or PR+) subtypes, endocrine therapies such as selective estrogen receptor modulators, selective estrogen receptor degraders, and aromatase inhibitors are generally used [3].

TNBC is an aggressive BC subtype with a poor prognosis and a high tendency to metastasize to distant tissues and organs [4]. Chemotherapy is the primary treatment option for aggressive tumors. Despite its widespread clinical use across BC subtypes, PTX exhibits considerable variability in treatment response, thereby limiting its suitability for all patients.

Paclitaxel (PTX), isolated from the plant *Taxus brevifolia* and used clinically as an anticancer agent, stabilizes microtubules, prevents their depolymerization, induces G2/M cell-cycle arrest, and triggers apoptotic cell death [5].

Genetic, phenotypic, and other variations significantly affect BC patients’ responses to the same drug. Unfortunately, there is no single cause of PTX resistance. It develops as cancer signaling becomes dysregulated, mutations accumulate, and epigenetic alterations occur, collectively leading to reduced therapeutic effectiveness and worse clinical outcomes [6]. Additionally, metabolism plays an important role in treatment response because changes in glycolysis, lipid uptake, and energy balance can affect how aggressively a tumor grows and how it responds to chemotherapy. These metabolic changes also influence apoptosis, proliferation, angiogenesis, invasion, epithelial–mesenchymal transition (EMT), and cellular senescence [7].

In view of this subtype-specific diversity, it is crucial to assess PTX response at the molecular level. In this work, we investigated the in vitro responses of PTX across molecularly distinct BC cell lines. We analyzed its effects on genes involved in cancer-related pathways, energy metabolism, and drug resistance. Apoptosis and cell-cycle responses were further characterized by flow cytometry, while the expression of key apoptosis- and cell-cycle-related proteins was evaluated by Western blotting. This integrated approach may provide insights into the reduced efficacy of PTX in aggressive subtypes and support the development of more effective therapeutic strategies, including combination-based approaches.

## 2. Results

### 2.1. Paclitaxel Reduces Cell Viability in Molecularly Distinct Breast Cancer Cell Lines

**Figure 1:**
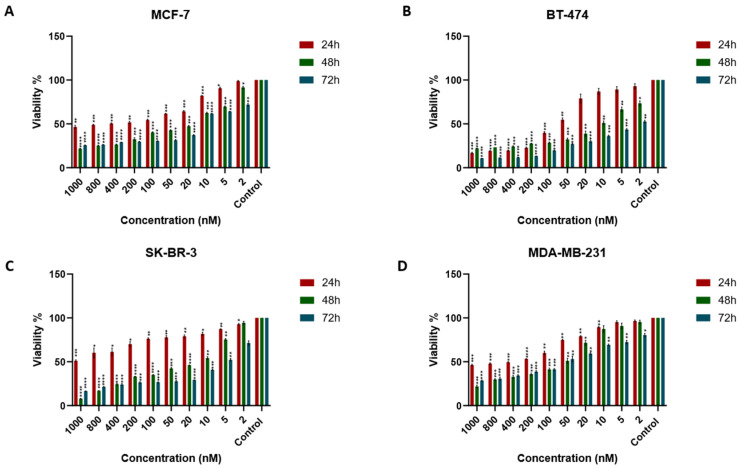
Time- and concentration-dependent effects of paclitaxel (PTX) on BC cell viability. BC cell lines (MCF-7, BT-474, SK-BR-3, and MDA-MB-231) were treated with PTX at concentrations ranging from 2 to 1000 nM for 24, 48, and 72 h. Cell viability was determined using the MTT assay and expressed as a percentage relative to untreated control cells, which were considered 100% viable. The effects of PTX on cell viability are presented as bar graphs for (**A**) MCF-7, (**B**) BT-474, (**C**) SK-BR-3, and (**D**) MDA-MB-231 cells. IC_50_ values were calculated for each cell line and treatment duration based on dose–response analysis. Data are presented as the mean ± SD of three independent experiments. Statistical analysis was performed using one-way ANOVA followed by Tukey’s post hoc test; *p* < 0.05 was considered statistically significant: * *p* < 0.05, ** *p* < 0.01, *** *p* < 0.001 and **** *p* < 0.0001.

The effect of PTX on BC cell viability was assessed using the MTT assay (Figure 1). As shown in Figure 1, PTX treatment reduced the viability of all BC cell lines in a concentration- and time-dependent manner. The IC_50_ values of PTX in MCF-7 cells after 24, 48, and 72 h of incubation were 16.95 ± 1.20 nM, 12.60 ± 0.90 nM, and 5.97 ± 0.60 nM, respectively (Figure 1A). In BT-474 cells, the IC_50_ values were 28.35 ± 1.12 nM, 5.09 ± 0.48 nM, and 3.46 ± 0.62 nM, respectively (Figure 1B). In SK-BR-3 cells, the IC_50_ values were 36.13 ± 1.85 nM, 16.09 ± 1.21 nM, and 3.92 ± 0.71 nM, respectively (Figure 1C). In MDA-MB-231 cells, the IC_50_ values were 39.42 ± 2.04 nM, 36.66 ± 2.31 nM, and 13.85 ± 1.67 nM, respectively (Figure 1D). These results suggest that PTX exerts concentration- and time-dependent inhibitory effects in BC cell lines.

Selectivity index (SI) analyses were performed by comparing the cytotoxic effects of PTX in BC cells with those in non-tumorigenic hTERT-HME1 epithelial cells. SI values greater than 1 indicate preferential toxicity towards cancer cells. In contrast, values close to or below 1 suggest limited selectivity and a narrow difference between anticancer activity and cytotoxicity in non-tumorigenic cells. Accordingly, higher SI values indicate a more favorable in vitro selectivity profile. To assess PTX selectivity for cancer cells, hTERT-HME1 cells were treated under identical experimental conditions. The IC_50_ values in hTERT-HME1 cells were calculated to be 20.32 ± 1.5 nM, 5.97 ± 0.4 nM, and 2.92 ± 0.3 nM at 24, 48, and 72 h, respectively. The selectivity index (SI), calculated as the IC_50_ in non-tumorigenic (hTERT-HME1) cells divided by that in cancer cells, was 0.47 for MCF-7, 1.17 for BT-474, 0.37 for SK-BR-3, and 0.16 for MDA-MB-231 cells at 48 h (see Table 1 for details).

### 2.2. Paclitaxel Induces Apoptotic Cell Death of Distinct Breast Cancer Cell Lines

**Figure 2:**
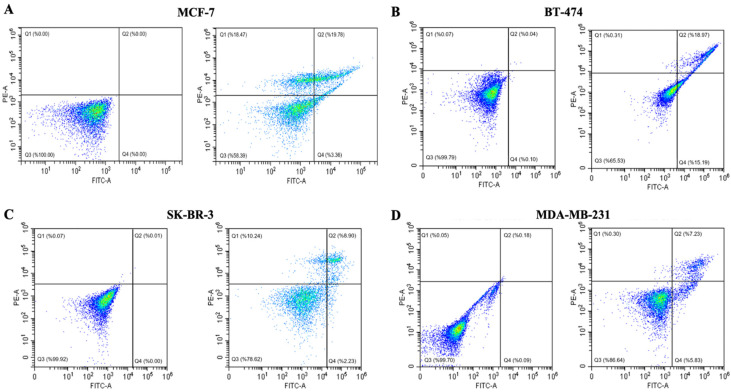
Apoptotic effects of paclitaxel (PTX) on BC cells determined by Annexin V-FITC/PI staining. BC cells (MCF-7, BT-474, SK-BR-3, and MDA-MB-231) were treated with PTX at their cell line-specific 48 h IC_50_ concentrations for 48 h. The applied PTX concentrations were 12.60 nM for MCF-7, 5.09 nM for BT-474, 16.09 nM for SK-BR-3, and 36.66 nM for MDA-MB-231 cells. After treatment, cells were stained with Annexin V-FITC/propidium iodide (PI), and apoptosis was evaluated by flow cytometry. Representative flow cytometry dot plots are shown for (**A**) MCF-7, (**B**) BT-474, (**C**) SK-BR-3, and (**D**) MDA-MB-231 cells. In each panel, the left plot shows untreated control cells, and the right plot shows PTX-treated cells. Quadrant analysis was interpreted as follows: Q1, necrotic cells (Annexin V−/PI+); Q2, late apoptotic/secondary necrotic cells (Annexin V+/PI+); Q3, viable cells (Annexin V−/PI−); and Q4, early apoptotic cells (Annexin V+/PI−). The color gradient represents event density, with blue indicating lower-density regions and green/yellow indicating higher-density regions.

The Annexin V-FITC/PI assay revealed a significant increase in the apoptotic cell population following PTX treatment in all BC cell lines. In MCF-7 cells treated with 12.60 nM PTX, the percentages of early- and late-stage apoptotic cells were 3.36% and 19.78%, respectively. In the BT-474 cell line treated with 5.09 nM PTX, early and late apoptosis rates increased to 15.19% and 18.97%, respectively, representing the highest apoptotic response among the tested cell lines. In SK-BR-3 cells treated with 16.09 nM PTX, the early and late apoptotic populations were 2.23% and 8.90%, respectively, whereas in MDA-MB-231 cells treated with 36.66 nM PTX, they were 5.83% and 7.23%, respectively. BT-474 cells exhibited a significantly higher apoptotic response than the other BC cell lines, demonstrating subtype-dependent sensitivity to PTX. Additionally, PTX treatment significantly increased the necrotic cell population in MCF-7 and SK-BR-3 cell lines (*p* < 0.0001) (Figure 2 and Table 2). Overall, the findings indicate that PTX induces the greatest apoptosis in BT-474 cells, whereas MDA-MB-231 cells exhibit lower apoptosis despite the highest IC50, suggesting decreased PTX sensitivity in the TNBC model (*p* < 0.0001) (Figure 2 and Table 2).

### 2.3. Paclitaxel Induces Subtype-Dependent Cell-Cycle Alterations in Molecularly Distinct Breast Cancer Cell Lines

**Figure 3:**
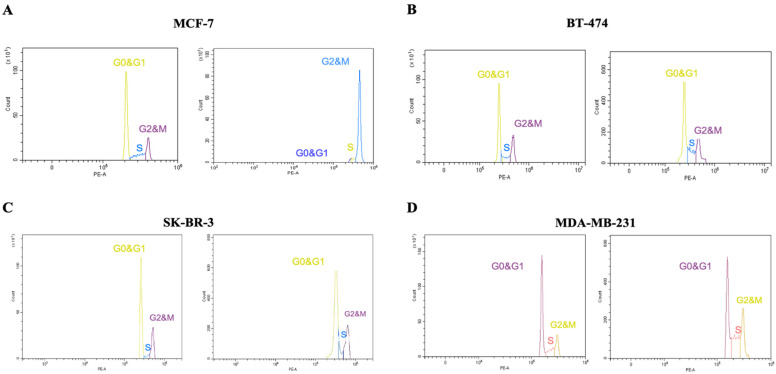
Effects of paclitaxel (PTX) on cell cycle distribution in BC cells. BC cells (MCF-7, BT-474, SK-BR-3, and MDA-MB-231) were treated with PTX at their cell line-specific 48 h IC_50_ concentrations for 48 h. The applied PTX concentrations were 12.60 nM for MCF-7, 5.09 nM for BT-474, 16.09 nM for SK-BR-3, and 36.66 nM for MDA-MB-231 cells. After treatment, cells were stained with propidium iodide (PI), and DNA content was analyzed by flow cytometry to determine the cell cycle distribution. Representative histograms are shown for (**A**) MCF-7, (**B**) BT-474, (**C**) SK-BR-3, and (**D**) MDA-MB-231 cells. In each panel, the left histogram represents untreated control cells, while the right histogram represents PTX-treated cells. G0/G1, S, and G2/M indicate the corresponding cell cycle phases.

**Figure 4:**
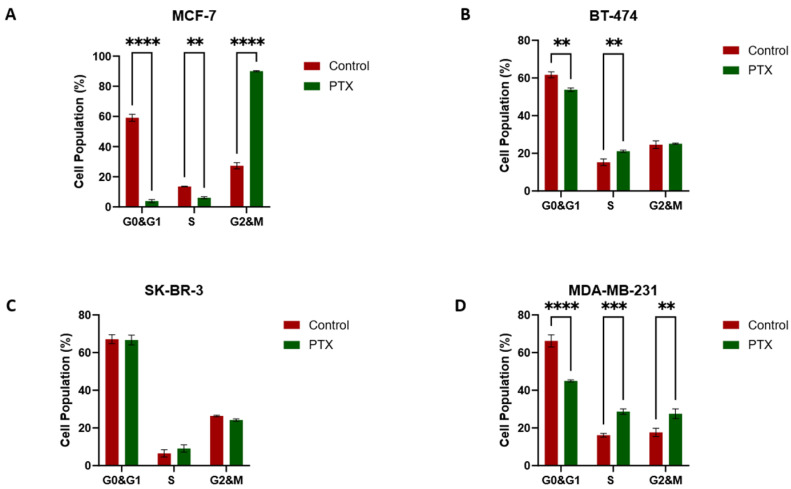
Quantitative analysis of cell-cycle phase distribution in BC cell lines following paclitaxel (PTX) treatment. MCF-7, BT-474, SK-BR-3, and MDA-MB-231 BC cells were treated with PTX at their respective 48 h IC_50_ concentrations for 48 h. The applied PTX concentrations were 12.60 nM for MCF-7 (**A**), 5.09 nM for BT-474 (**B**), 16.09 nM for SK-BR-3 (**C**), and 36.66 nM for MDA-MB-231 (**D**) cells. After treatment, cells were stained with propidium iodide (PI), and DNA content was analyzed by flow cytometry. The percentages of cells in the G0/G1, S, and G2/M phases were quantified and compared between untreated control and PTX-treated cells. Data are presented as the percentage of the total cell population in each phase. Statistical analysis was performed using one-way ANOVA followed by Tukey’s post hoc test; *p* < 0.05 was considered statistically significant: ** *p* < 0.01, *** *p* < 0.001, **** *p* < 0.0001.

Cell-cycle analysis showed that PTX altered cell-cycle distribution in four BC cell lines in distinct ways. In MCF-7 cells, the G2/M population increased sharply from 27.67% to 90.26%, while the G0/G1 population decreased from 57.91% to 3.14%, consistent with a strong G2/M arrest. The response in BT-474 cells was minimal, with the G2/M population remaining nearly constant (26.05% vs. 25.34%), suggesting limited PTX sensitivity at the cell-cycle level. SK-BR-3 cells showed a pattern similar to that of BT-474 cells, with the G2/M population decreasing slightly from 26.11% to 23.75%. PTX treatment in the TNBC cell line MDA-MB-231 resulted in moderate cell redistribution, with a decrease in the G0/G1 population from 63.99% to 45.39% and simultaneous increases in the S phase population (from 16.82% to 27.68%) and G2/M population (from 19.19% to 26.36%), which suggests partial disruption to the cell cycle rather than overt arrest. Overall, these findings suggest that PTX predominantly arrests cells in the G2/M population in MCF-7 cells, while exerting limited or moderate effects on other BC cell lines (Figure 3 and Figure 4).

### 2.4. RT-qPCR and Gene Enrichment

**Figure 5:**
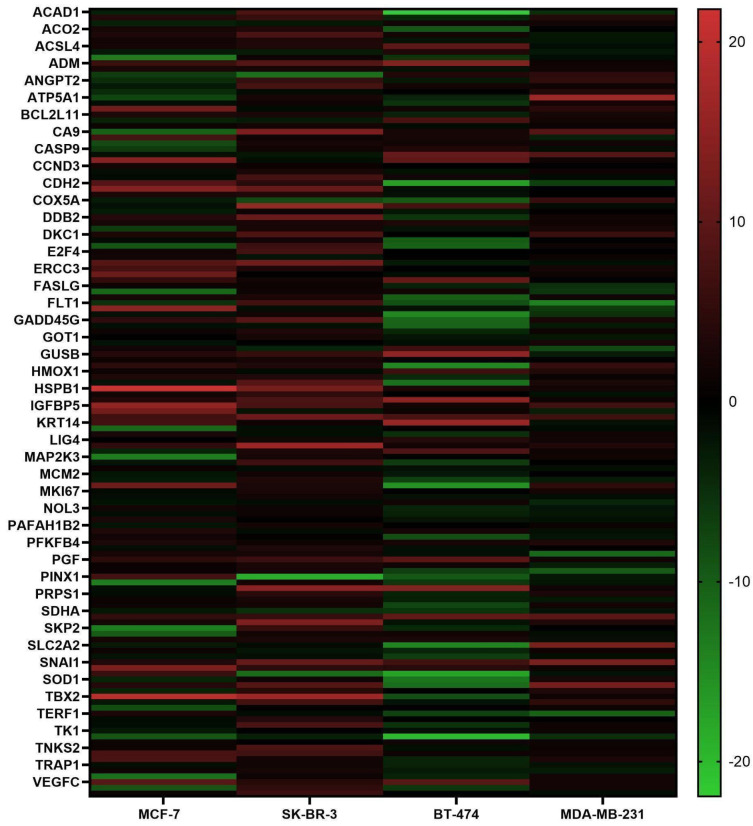
Heatmap showing PTX-induced differential gene expression patterns in BC cell lines. MCF-7, SK-BR-3, BT-474, and MDA-MB-231 BC cells were treated with their respective 48 h IC_50_ concentrations of paclitaxel (PTX) for 48 h. The applied PTX concentrations were 12.60 nM for MCF-7, 16.09 nM for SK-BR-3, 5.09 nM for BT-474, and 36.66 nM for MDA-MB-231 cells. Differential gene expression levels were determined by quantitative RT-qPCR and calculated relative to untreated control cells using the 2^−ΔΔCt^ method after normalization to internal reference gene(s). The heatmap represents relative fold-change values of selected genes associated with apoptosis, angiogenesis, DNA damage/repair, cell-cycle regulation, migration, and metabolic pathways. Red indicates increased gene expression, green indicates decreased gene expression, and black indicates no or minimal change compared with the untreated control. The heatmap was generated using GraphPad Prism version 11.0.0.

**Figure 6:**
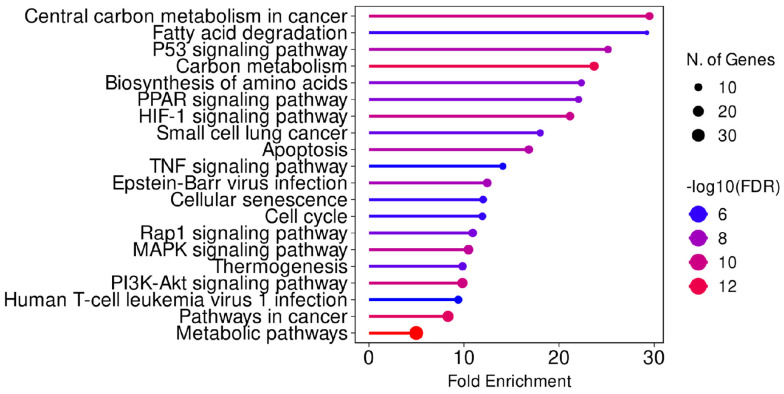
MCF-7 cells were treated with the cell line-specific 48 h IC_50_ concentration of paclitaxel (PTX; 12.60 nM) for 48 h. Differentially expressed genes were determined by RT-qPCR and calculated relative to untreated control cells using the 2^−ΔΔCt^ method. Genes showing ≥2-fold upregulation or ≤0.5-fold downregulation were included in KEGG pathway enrichment analysis using ShinyGO 0.82. The top 20 enriched KEGG pathways are shown and ranked according to fold enrichment. Enriched pathways primarily reflect PTX-associated alterations in cancer-related signaling, metabolic regulation, stress response, and cell death.

The effects of PTX treatment on genes associated with cancer-related signaling pathways, energy metabolism, and drug resistance were assessed by RT-qPCR. RNA isolation was performed after PTX treatment, and RT-qPCR was carried out using the array lists in Supplementary Tables S1 and S2. The 2^−ΔΔCt^ method was used to determine relative gene expression levels. PTX treatment induced differential, cell-line-dependent changes in gene expression in BC cells. (Supplementary Tables S3 and S12; Figure 5 and Figure 6).

**Figure 7:**
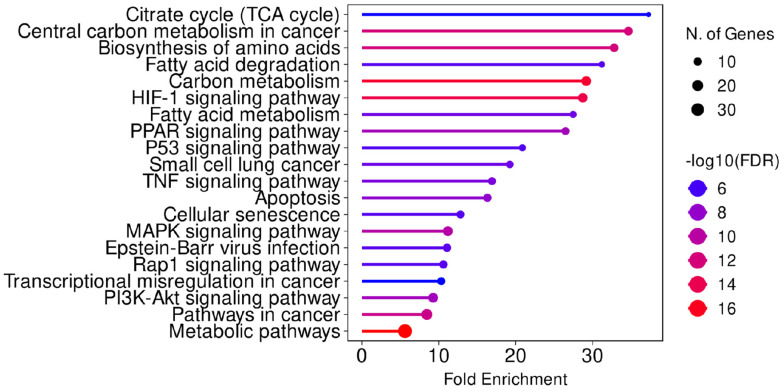
SK-BR-3 cells were treated with the cell line-specific 48 h IC_50_ concentration of paclitaxel (PTX; 16.09 nM) for 48 h. Differentially expressed genes were determined by RT-qPCR and calculated relative to untreated control cells using the 2^−ΔΔCt^ method. Genes showing ≥2-fold upregulation or ≤0.5-fold downregulation were included in KEGG pathway enrichment analysis using ShinyGO 0.82. The top 20 enriched KEGG pathways are shown and ranked according to fold enrichment. The enriched pathways indicate PTX-associated modulation of metabolic pathways, cancer-related signaling, hypoxia- and stress-associated responses, and cell survival mechanisms in HER2-enriched SK-BR-3 cells.

**Figure 8:**
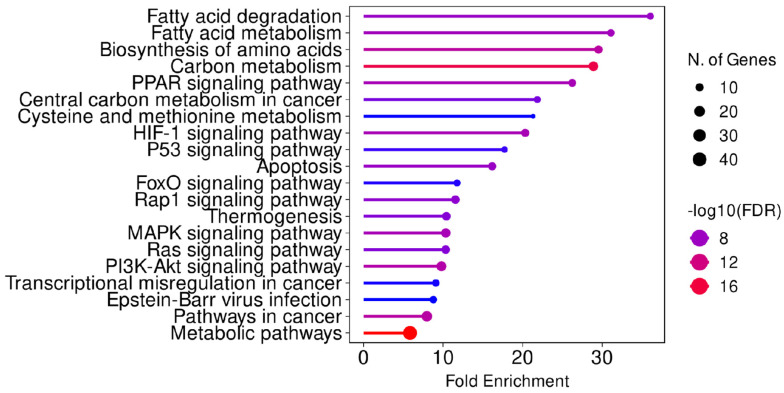
BT-474 cells were treated with the cell line-specific 48 h IC_50_ concentration of paclitaxel (PTX; 5.09 nM) for 48 h. Differentially expressed genes were determined by RT-qPCR and calculated relative to untreated control cells using the 2^−ΔΔCt^ method. Genes showing ≥2-fold upregulation or ≤0.5-fold downregulation were included in KEGG pathway enrichment analysis using ShinyGO 0.82. The top 20 enriched KEGG pathways are shown and ranked according to fold enrichment. The enrichment profile reflects PTX-induced regulation of cancer-associated pathways, energy metabolism, lipid-related metabolism, and stress-response signaling in BT-474 cells.

**Figure 9:**
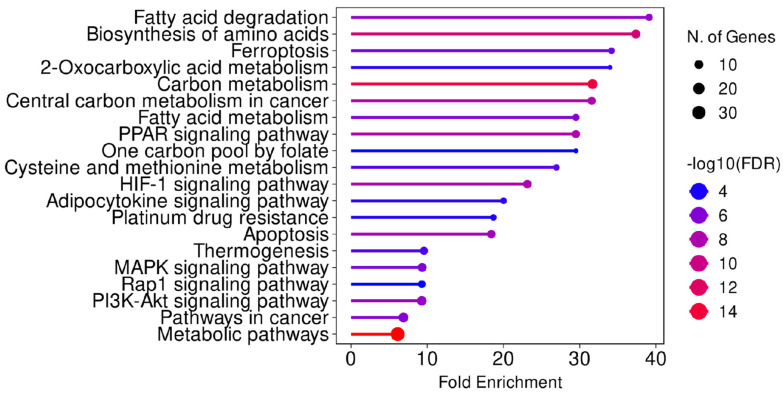
MDA-MB-231 cells were treated with the cell line-specific 48 h IC_50_ concentration of paclitaxel (PTX; 36.66 nM) for 48 h. Differentially expressed genes were determined by RT-qPCR and calculated relative to untreated control cells using the 2^−ΔΔCt^ method. Genes showing ≥2-fold upregulation or ≤0.5-fold downregulation were included in KEGG pathway enrichment analysis using ShinyGO 0.82. The top 20 enriched KEGG pathways are shown and ranked according to fold enrichment. The enriched pathways suggest PTX-associated changes in metabolic reprogramming, cancer-related signaling, stress adaptation, and survival-related pathways in triple-negative MDA-MB-231 cells.

Genes exhibiting differential expression, defined as ≥2-fold upregulation or ≤0.5-fold downregulation, were selected for KEGG pathway enrichment analysis using ShinyGO 0.82. While the relative rankings of enriched pathways varied across cell lines, the overall enrichment patterns were largely comparable (Figure 6, Figure 7, Figure 8 and Figure 9 and Supplementary Tables S4–S7). PTX treatment was associated with several enriched pathways across the molecularly distinct BC cell line models. These pathways include central carbon metabolism in cancer, fatty acid degradation, amino acid biosynthesis, and carbon metabolism, indicating an effect on cancer-related metabolic reprogramming. Common enrichment of signaling pathways also suggests that PTX response involves regulation of lipid, hypoxia-related, survival, stress, and cell death pathways. These results support the presence of both common and cell line-dependent responses to PTX exposure in BC cells.

### 2.5. Gene–Metabolite Interactions

**Figure 10:**
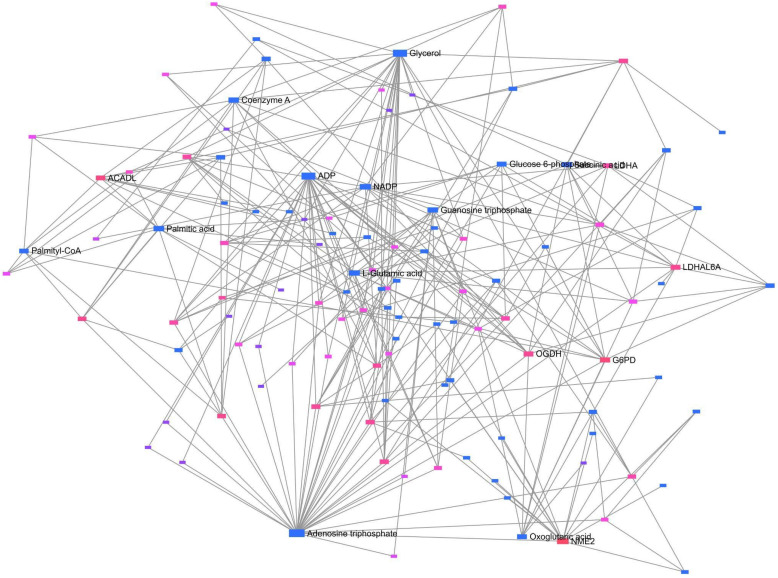
MCF-7 cells were treated with the cell line-specific 48 h IC_50_ concentration of paclitaxel (PTX; 12.60 nM) for 48 h. Differentially expressed genes were determined by RT-qPCR relative to untreated control cells using the 2^−ΔΔCt^ method. Genes showing differential expression were analyzed using Enrichr together with the Metabolomics Workbench Metabolites 2022 database, and the resulting database-derived gene–metabolite associations were visualized using MetaboAnalyst 6.0. Pink rectangles indicate genes, blue rectangles indicate metabolites, and connecting edges indicate predicted gene–metabolite associations. The network highlights functional modules associated with glycolysis/pentose phosphate metabolism, lipid/fatty acid metabolism, TCA/anaplerotic metabolism, and nucleotide/energy metabolism.

**Figure 11:**
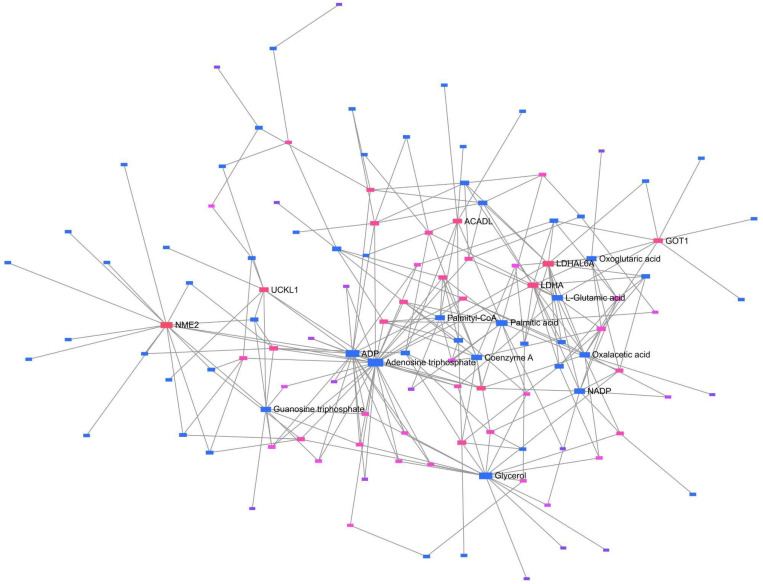
SK-BR-3 cells were treated with the cell line-specific 48 h IC_50_ concentration of paclitaxel (PTX; 16.09 nM) for 48 h. Differentially expressed genes were determined by RT-qPCR relative to untreated control cells using the 2^−ΔΔCt^ method. Genes showing differential expression were analyzed using Enrichr together with the Metabolomics Workbench Metabolites 2022 database, and the resulting database-derived gene–metabolite associations were visualized using MetaboAnalyst 6.0. Pink rectangles indicate genes, blue rectangles indicate metabolites, and connecting edges indicate predicted gene–metabolite associations. The network highlights functional modules associated with TCA/anaplerotic metabolism, glycolysis/pentose phosphate metabolism, lipid/fatty acid metabolism, and nucleotide/energy metabolism.

**Figure 12:**
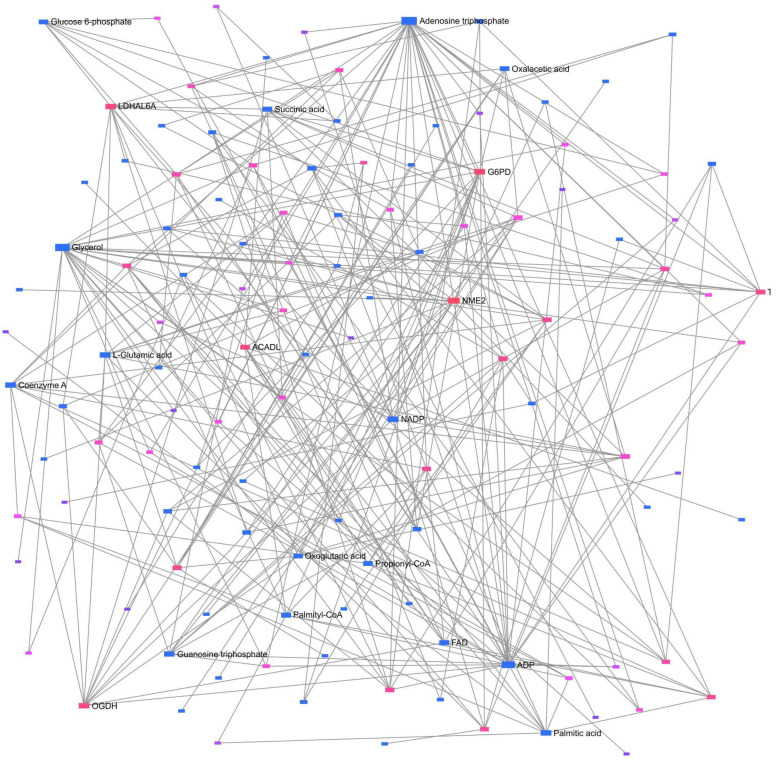
BT-474 cells were treated with the cell line-specific 48 h IC_50_ concentration of paclitaxel (PTX; 5.09 nM) for 48 h. Differentially expressed genes were determined by RT-qPCR relative to untreated control cells using the 2^−ΔΔCt^ method. Genes showing differential expression were analyzed using Enrichr together with the Metabolomics Workbench Metabolites 2022 database, and the resulting database-derived gene–metabolite associations were visualized using MetaboAnalyst 6.0. Pink rectangles indicate genes, blue rectangles indicate metabolites, and connecting edges indicate predicted gene–metabolite associations. The network highlights functional modules associated with lipid/fatty acid metabolism, glycolytic/lactate-associated metabolism, TCA/anaplerotic metabolism, and nucleotide/energy metabolism.

**Figure 13:**
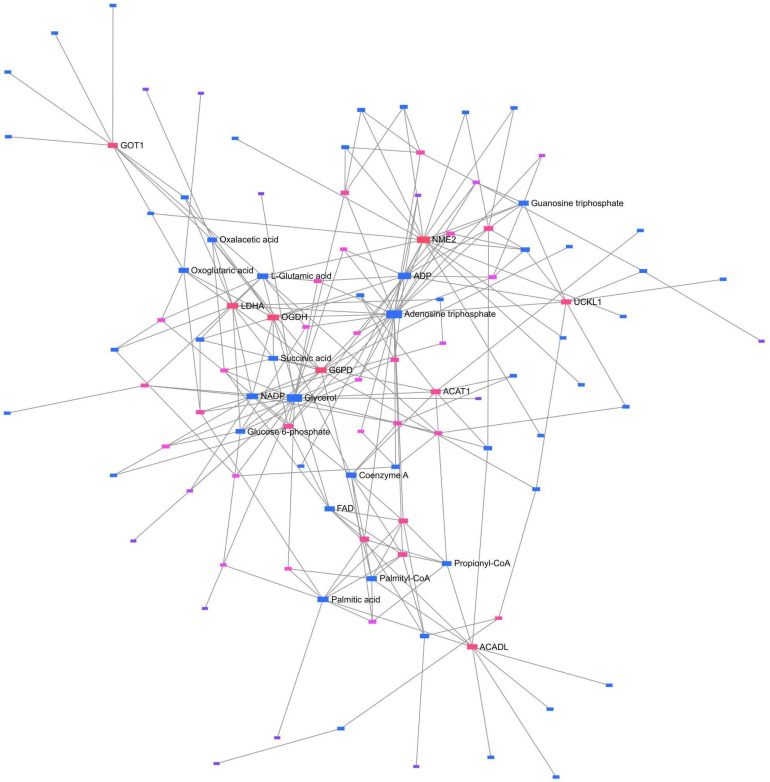
MDA-MB-231 BC cells were treated with the cell line-specific 48 h IC_50_ concentration of paclitaxel (PTX; 36.66 nM) for 48 h. Differentially expressed genes were determined by RT-qPCR relative to untreated control cells using the 2^−ΔΔCt^ method. Genes showing differential expression were analyzed using Enrichr together with the Metabolomics Workbench Metabolites 2022 database, and the resulting database-derived gene–metabolite associations were visualized using MetaboAnalyst 6.0. Pink rectangles indicate genes, blue rectangles indicate metabolites, and connecting edges indicate predicted gene–metabolite associations. The network highlights functional modules associated with glycolysis/pentose phosphate metabolism, TCA/anaplerotic metabolism, lipid/fatty acid metabolism, and nucleotide/energy metabolism.

To identify metabolite-associated gene signatures after PTX treatment, differentially expressed genes were analyzed using Enrichr together with the Metabolomics Workbench Metabolites 2022 database (Supplementary Tables S8–S11). The resulting gene–metabolite associations were then visualized using MetaboAnalyst 6.0. It should be noted that the gene–metabolite interaction networks shown in Figure 10, Figure 11, Figure 12 and Figure 13 do not directly display KEGG pathway terms. Rather, they show predicted gene–metabolite associations that correspond to the metabolic components of the enriched pathways identified in Figure 6, Figure 7, Figure 8 and Figure 9. Therefore, the core cancer-related metabolic pathways detected by KEGG enrichment are represented in (Figure 10, Figure 11, Figure 12 and Figure 13) as functional gene–metabolite modules rather than as pathway names.

In MCF-7 cells, database-derived gene–metabolite enrichment analysis revealed predicted associations among the metabolites glycerol, glucose-6-phosphate, coenzyme A, ADP, ATP, NADP, palmitic acid, and palmitoyl-CoA, and the genes *LDHAL6A*, *ACADL*, *OGDH*, *G6PD*, and *NME2* (Figure 10). These associations indicate a network centered on glycolysis/pentose phosphate metabolism, lipid utilization, and nucleotide/energy metabolism. The presence of glucose-6-phosphate, *G6PD*, NADP, ATP/ADP, and palmitoyl-CoA suggests that the KEGG-enriched central carbon metabolism and fatty acid-related pathways are reflected in this subtype through metabolic nodes involved in redox balance, ATP turnover, and lipid remodeling.

In SK-BR-3 cells, database-derived gene–metabolite enrichment analysis identified predicted associations linking succinic acid, oxaloacetic acid, ATP, glucose-6-phosphate, glycerol, L-glutamic acid, coenzyme A, oxoglutaric acid, palmitic acid, FAD, and ADP metabolites to the *OGDH*, *LDHAL6A*, *G6PD*, *NME2*, and *TK1* genes (Figure 11). Compared with MCF-7 cells, SK-BR-3 cells showed a more prominent TCA/anaplerotic module, including succinic acid, oxaloacetic acid, oxoglutaric acid, *OGDH*, and L-glutamic acid. This suggests that PTX response in HER2-enriched SK-BR-3 cells involves mitochondrial carbon flux, redox-linked metabolism, and nucleotide metabolism.

In BT-474 cells, database-derived gene–metabolite enrichment analysis identified predicted associations linking oxoglutaric acid, L-glutamic acid, oxaloacetic acid, palmitic acid, palmitoyl-CoA, ADP, ATP, and glycerol metabolites to the *NME2*, *UCKL1*, *ACADL*, *LDHA*, *LDHAL6A*, and *GOT1* genes (Figure 12). This network showed combined involvement of lipid/fatty acid metabolism, glycolytic/lactate-associated metabolism, and TCA-linked anaplerotic metabolism. The presence of *GOT1* and oxaloacetic acid/oxoglutaric acid suggests a stronger connection between amino acid metabolism and mitochondrial carbon handling in BT-474 cells.

In MDA-MB-231 cells, database-derived gene–metabolite enrichment analysis identified predicted associations linking GTP, ADP, ATP, NADP, glycerol, palmitoyl-CoA, oxaloacetic acid, oxoglutaric acid, succinyl-CoA, glucose-6-phosphate, and palmitic acid metabolites with the *GOT1*, *LDHA*, *OGDH*, *NME2*, *UCKL1*, *ACADL*, and *ACAT1* (Figure 13). This network indicates a more complex metabolic adaptation involving energy metabolism, nucleotide metabolism, lipid remodeling, glycolysis/pentose phosphate metabolism, and TCA/anaplerotic metabolism. The broader network structure in TNBC cells and MDA-MB-231 cells may reflect the metabolic plasticity of the triple-negative subtype under PTX-induced stress.

Collectively, these results show that the common KEGG-enriched cancer-related metabolic pathways are also represented in the gene–metabolite interaction networks, but at the level of specific metabolite–gene modules. The revised Figure 10, Figure 11, Figure 12 and Figure 13 now highlight these modules as glycolysis/pentose phosphate metabolism, TCA/anaplerotic metabolism, lipid/fatty acid metabolism, and nucleotide/energy metabolism. This visualization clarifies both the shared metabolic core and the subtype-specific differences in PTX response.

### 2.6. Drug Resistance

**Figure 14:**
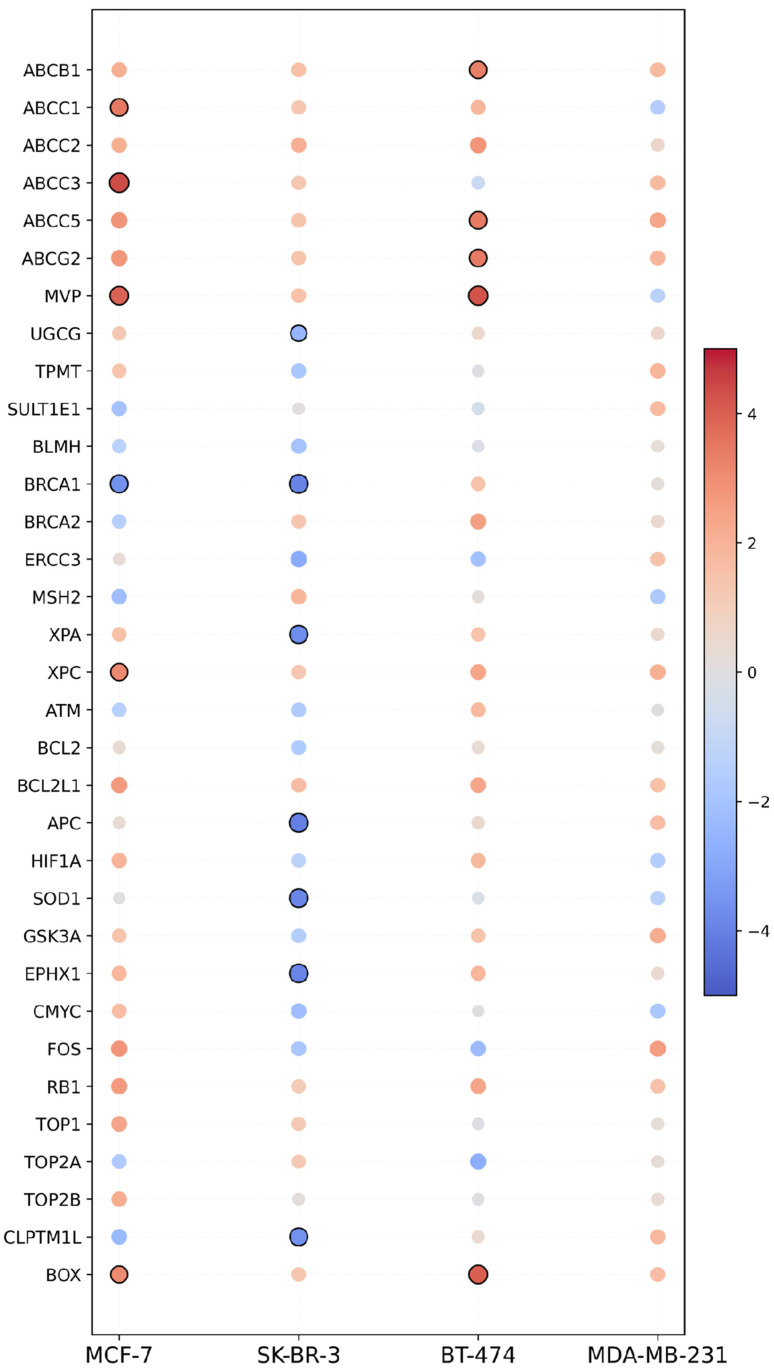
Differential expression of drug resistance-related genes in PTX-treated BC cells. BC cells (MCF-7, BT-474, SK-BR-3, and MDA-MB-231) were treated with PTX at their cell line-specific 48 h IC_50_ concentrations for 48 h. Drug resistance-related gene expression was evaluated by RT-qPCR using the drug resistance array listed in Supplementary Table S2, and fold-change values were calculated relative to untreated control cells using the 2^−ΔΔCt^ method. Bubble color represents the direction and magnitude of fold-change values, with red indicating upregulation and blue indicating downregulation. Bubble size corresponds to the absolute fold-change magnitude. Black outlines indicate statistically significant changes compared with untreated control cells.

RT-qPCR analysis of drug resistance-related genes was performed using the drug resistance array listed in Supplementary Table S2. Following PTX exposure, subtype-specific changes were observed in genes associated with multidrug resistance, chemotherapy response, DNA damage response, and cellular survival mechanisms. Notable expression changes were observed in the ATP-binding cassette (ABC) transporter family and in several genes involved in chemotherapeutic stress-response pathways. PTX induced the expression of resistance-linked genes at varying levels across subtypes. ABC transporter genes were upregulated in luminal and HER2-rich models, whereas various other resistance-associated genes were downregulated in MDA-MB-231 cells. These findings demonstrate transcriptional remodeling of drug resistance pathways after PTX exposure, but do not prove PTX resistance (Supplementary Table S12 and Figure 14).

### 2.7. Western Blot

**Figure 15:**
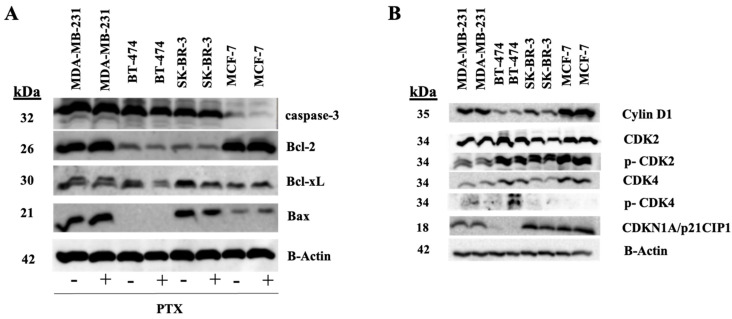
Effects of paclitaxel (PTX) on apoptosis- and cell cycle-related proteins in BC cells. BC (MCF-7, BT-474, SK-BR-3, and MDA-MB-231) cells were treated with PTX at their cell line-specific 48 h IC_50_ concentrations for 48 h. Protein expression levels were evaluated by Western blot analysis. (**A**) Representative Western blot images showing the levels of apoptosis-related proteins, including caspase-3, Bcl-2, Bcl-xL, and Bax. (**B**) Representative Western blot images showing the levels of cell cycle-related proteins, including cyclin D1, CDK2, phospho-CDK2, CDK4, phospho-CDK4, and CDKN1A/p21CIP1. Untreated cells (−) serve as controls, while PTX-treated cells are indicated as (+). β-Actin was used as the loading control. The expected molecular weights of the detected proteins are indicated on the left.

Western blotting was performed to evaluate the effects of PTX on the cell cycle and apoptotic proteins. In the BT-474 cells, the decrease in anti-apoptotic Bcl-2 and Bcl-xL protein levels was consistent with the highest total apoptotic population detected by Annexin V-FITC/PI analysis. In MCF-7 cells, the decrease in phosphorylated CDK2 and CDK4 and the increase in CDKN1A/p21CIP1 expression were consistent with the marked G2/M accumulation observed in cell cycle analysis. On the other hand, SK-BR-3 and MDA-MB-231 cells exhibited more heterogeneous changes in protein expression, supporting the idea that the PTX-induced checkpoint response is weaker or less homogeneous in these models. Overall, these findings demonstrate that PTX-induced apoptosis and cell cycle regulation differ significantly among molecularly distinct BC cell lines (Figure 15).

## 3. Discussion

Paclitaxel (PTX), a natural chemotherapeutic agent isolated from *Taxus* species, remains one of the most widely used chemotherapeutic agents in BC treatment. Its anticancer activity is primarily attributed to the stabilization of microtubules through interaction with β-tubulin on their luminal surface, thereby disrupting normal microtubule dynamics, impairing mitotic progression, and ultimately inducing cytotoxic stress in rapidly proliferating cancer cells [7]. BC is a highly heterogeneous disease, and its molecular subtypes differ substantially in biological behavior, treatment sensitivity, and mechanisms of resistance. In the present study, the cytotoxic, apoptotic, and molecular effects of PTX were evaluated across molecularly distinct BC cell lines representing luminal A (MCF-7), luminal B (BT-474), HER2+ (SK-BR-3), and TNBC (MDA-MB-231) subtypes, revealing pronounced subtype-specific differences in drug response. PTX reduced cell viability in a concentration- and time-dependent manner in BC cells, with IC_50_ values in the nanomolar range. Among the BC cell lines, MDA-MB-231 exhibited higher IC_50_ values, indicating lower PTX sensitivity than the luminal and HER2-enriched models [8]. Overall, these findings showed that luminal and HER2+ cell lines were more sensitive to PTX than the TNBC cell line. Overall, these findings suggest that luminal and HER2+ cell lines were more sensitive to PTX than the TNBC cell line, consistent with previous reports showing variable taxane responses among BC subtypes [9]. However, PTX also exhibited cytotoxicity against non-tumorigenic hTERT-HME1 epithelial cells, indicating that selectivity remains a key limitation. Therefore, further studies should evaluate strategies that improve the therapeutic window of PTX while preserving its antitumor activity.

Apoptosis increased across all examined subtypes after PTX treatment, but the magnitude and nature of the response varied by cell line. In the BT-474 cell line, the combination of early and late apoptosis (34.16%) showed the strongest effect, indicating greater sensitivity in the HER2+/luminal-B model. Based on transcriptional data, upregulation of BAX and *CASP9* supports a mitochondrial apoptotic phenotype, while increased *APAF1* and *CASP7* support mitochondria-mediated caspase activation. Protein-level data also suggest that PTX treatment shifted BT-474 cells toward a less anti-apoptotic state, as shown by decreased Bcl-2 and Bcl-xL expression. Although Bax protein expression was weak, the reduction in anti-apoptotic Bcl-2 family members was consistent with the flow cytometry and RT-qPCR findings. In MCF-7 cells, increased *CASP2* expression suggests that caspase-2-related signaling may contribute to PTX-mediated cytotoxicity. Previous studies have shown that PTX concentrations above approximately 12 nM are required to suppress proliferation and induce G2/M arrest, whereas lower concentrations primarily promote apoptotic cell death in BC cells [10,11]. In the present study, only minor changes were observed in Bcl-2 family proteins, with a slight increase in Bax and minimal changes in Bcl-2 and Bcl-xL. Caspase-3 protein expression was very low, consistent with the literature and with the known caspase-3 deficiency phenotype of MCF-7 cells. These findings suggest that apoptosis in MCF-7 cells may depend more on alternative signaling mechanisms than on canonical caspase-3 activation. SK-BR-3 cells also showed an evident apoptotic response. PTX treatment increased Bax protein expression and decreased Bcl-2 and Bcl-xL levels, suggesting a shift in the Bcl-2 balance toward apoptosis.

The protein-level findings, flow cytometry results, and transcriptional changes were consistent with each other. The detected caspase-3 band likely represents pro-caspase-3, suggesting apoptosis-related signaling rather than direct caspase-3 activation. In MDA-MB-231 cells, PTX modulated apoptosis-related genes but induced a weaker apoptotic response, resulting in a total apoptotic cell death of 13.06%. Upregulation of *CASP7* and *BCL2L11*, together with suppression of *XIAP* and *BCL2*, indicates intrinsic apoptotic signaling. At the protein level, Bax increased, and Bcl-xL slightly decreased, while Bcl-2 and total/pro-caspase-3 remained largely unchanged. These findings suggest a shift toward pro-apoptotic signaling without clear full activation of the mitochondrial caspase cascade. Previous studies have shown that PTX can induce intrinsic mitochondrial apoptosis through BAX activation, cytochrome c release, and initiation of the caspase cascade [10,11]. Therefore, the weaker apoptotic response in MDA-MB-231 cells may be associated with impaired apoptotic signaling or alternative survival mechanisms frequently reported in aggressive TNBC models.

The cell cycle is primarily controlled by cyclin-dependent kinases (CDKs), and changes in CDK activity resulting from genetic and epigenetic alterations can lead to abnormal cell division and tumor formation [12]. CCND2 and CCND3 drive the G1/S transition through CDK4/6 activation [13]. Furthermore, CDC20 is key to the activity of APC/C and is found at high levels in many aggressive cancers, including BC, and is linked to poor prognosis [14,15]. In the present study, luminal BC cell lines exhibited marked downregulation of *CCND2*, *CCND3*, and *CDC20*, accompanied by accumulation of cells in the G2/M phase following PTX treatment. In luminal A MCF-7 cells, PTX treatment increased the G2/M population by 82.59% while reducing the G0/G1 population by 54.77% compared with the control group. Western blot data from the study support the idea that PTX modulates cell-cycle regulatory proteins in a cell-line-dependent manner; the most pronounced checkpoint-related response was observed in MCF-7 cells, while more heterogeneous responses were observed in BT-474, SK-BR-3, and MDA-MB-231 cells. PTX treatment showed a considerable reduction in cyclin D1 in BT-474 cells, while modulation of *CDK2* and *CDK4* was more heterogeneous, indicating disruption of G1/S-associated cell-cycle regulation rather than complete checkpoint suppression. The low CDKN1A/p21CIP1 protein expression suggests that the observed S-phase abundance is less likely to reflect p21-CDK suppression. Following PTX treatment, there were relatively minor changes in the levels of cyclin D1, CDK2, CDK4, phosphorylated CDK2 and CDK4, and CDKN1A/p21CIP1 in SK-BR-3 cells. These small changes in protein levels suggest that PTX had a weaker effect on the regulation of cell-cycle checkpoints in this HER2-enriched model. MDA-MB-231 cells redistributed across both S and G2/M phases. While cyclin D1 and total CDK2 levels were largely conserved, phosphorylated CDK4 levels were decreased, and CDKN1A/p21CIP1 showed limited change. This mixed protein profile is consistent with a shift across both S and G2/M phases, suggesting that PTX may affect multiple cell-cycle checkpoints without a uniform arrest pattern. The proliferation-marker data follow the same pattern. Decreased expression of *STMN1*, *AURKA*, and *MKI67* in luminal cell lines suggests suppressed proliferation. Conversely, increased expression of these genes in MDA-MB-231 cells suggests continued division and decreased PTX sensitivity.

DNA damage response (DDR) pathways are essential for maintaining genomic integrity, and their dysregulation contributes to BC development, therapeutic resistance, and disease progression [16]. Genes involved in DNA repair, oxidative stress, and proteostasis, including *GADD45G*, *PPP1R15A*, and *HSP90AB1*, are therefore important mediators of cellular stress responses [17]. *GADD45G* has been associated with cell-cycle arrest, apoptosis, DNA repair, and tumor-suppressive activity in BC, and is also linked to MAPK-related signaling regulation [18,19]. In the present study, PTX treatment increased *GADD45G* expression in HER2+ and TNBC cells, suggesting activation of DNA damage- and stress-related signaling pathways. PTX also altered the expression of stress- and metabolism-related genes, including *PPP1R15A*, *DDIT3*, *FOS*, *TPI1*, *LDHA*, *IGFBP3*, *STMN1*, and *AURKA*. Since *PPP1R15A* is involved in growth arrest, genotoxic stress, nutrient deprivation, and apoptosis-related signaling, these changes suggest that PTX may modulate p53-associated stress responses, DNA damage signaling, and metabolic adaptation pathways in BC cells [20,21].

HSP90 family proteins are closely associated with cancer progression, poor prognosis, metabolic adaptation, and therapy resistance in BC. TRAP1 regulates mitochondrial homeostasis and promotes metabolic reprogramming by suppressing oxidative phosphorylation and enhancing glycolytic activity, thereby reducing ROS production and conferring resistance to apoptosis [22]. HSP90AB1 overexpression has also been linked to tumor formation, angiogenesis, invasion, and unfavorable clinical outcomes in multiple cancer types [23]. HSPB1, also known as heat shock protein 27, protects cells against stress and regulates apoptosis, metastasis, and therapy-induced resistance in malignant cells [24]. Increased HSPB1 expression has also been associated with EMT through regulation of EMT-associated transcription factors, including *SNAI1* and *PRRX1* [25]. In the present study, PTX treatment altered HSP-related genes, including *TRAP1*, *CDC37*, *CDK4*, *HSP90AB1*, *HSP90AB4P*, and *HSPB1*, across BC subtypes. Increased *HSP90AB1* expression in luminal A and HER2+ BC cells was accompanied by elevated expression of angiogenesis-related genes, including *KDR*, *PGF*, and *VEGFC*. Similarly, increased HSPB1 expression was accompanied by elevated expression of EMT-related genes, including *SNAI1*, *SNAI2*, and *SOX10*. These findings suggest that PTX modulates HSP-associated signaling networks in a subtype-dependent manner and may contribute to stress adaptation, angiogenesis, EMT-related processes, and metabolic responses in BC cells.

Therapy-induced senescence is an important response to chemotherapeutic stress and may result from DNA damage, prolonged mitotic arrest, telomere dysfunction, and sustained stress signaling [26]. In BC cells, CDK4/6 dysregulation, TP53-associated signaling, and telomere instability are considered key drivers of senescence induction [27,28]. Chemotherapy-induced DNA damage may also promote telomere shortening and replicative senescence, whereas cancer cells can escape this process through telomerase reactivation [29]. In line with this, telomerase inhibitors such as BIBR1532 and GRN163L have been shown to suppress cancer cell proliferation and promote senescence-associated responses [30]. In the present study, PTX altered the expression of genes associated with telomere regulation, cell-cycle control, apoptosis, and senescence-related signaling, including *TMSB2*, *PINX1*, *TINF2*, *TERF1*, *TEP1*, *TERF2IP*, *TNKS*, *CCND2*, *CDK4*, *TP53*, *CASP9*, *BCL2L1*, *BCL2L11*, and *MAP2K1*. These findings suggest that PTX may contribute to senescence-associated growth arrest through subtype-dependent regulation of telomere maintenance, cell-cycle progression, and stress-response pathways in BC cells.

Metabolic reprogramming, characterized by increased glucose metabolism and aerobic glycolysis, supports cancer cell proliferation and survival [31]. Hypoxia-inducible factors (HIFs) promote tumor adaptation to low-oxygen conditions by regulating glycolysis- and angiogenesis-related genes, including *LDHA*, *PKM2*, *GLUT1*, and *PDK1*, while PI3K/Akt/mTOR signaling further enhances HIF-mediated glycolytic metabolism [32,33]. In the present study, PTX altered the expression of metabolism-related genes across BC subtypes. Downregulation of glycolysis-associated genes, including *LDHA*, *HK*, *PDK*, *LDHAL6A*, *SLC2A1*, and *PFKL*, particularly in luminal and HER2+ BC cells, suggests reduced HIF-associated glycolytic activity and partial suppression of Warburg-like metabolism. In contrast, partial preservation of glycolysis-associated gene expression in TNBC cells may reflect greater metabolic flexibility under therapeutic stress, consistent with previous studies showing that TNBC cells can switch between metabolic programs to survive chemotherapy [34]. PTX also modulated genes involved in MYC/TP53-associated metabolic regulation, the pentose phosphate pathway, redox homeostasis, and lipid metabolism. Reduced MYC expression in PTX-treated TNBC cells may indicate decreased transcriptional support for glycolysis, whereas increased *TP53* expression may suggest a possible shift toward oxidative metabolism; however, transcriptomic data alone are insufficient to confirm this directly. Altered expression of *G6PD*, *TKTL1*, and *PRPS1* suggests altered redox regulation, while reduced *G6PD* expression may indicate reduced NADPH-generating capacity. In HER2+ cells, changes in *ACLY*, *ACADL*, *CPT1C*, and *HMGCS1* suggest subtype-specific regulation of lipid metabolism. Overall, these findings indicate that PTX affects glycolytic, redox, and lipid metabolism-related gene expression in a subtype-dependent manner, which may contribute to differential treatment responses among BC cell lines.

Chemoresistance is still one of the most significant problems in BC treatment, tied to poor response, recurrence, metastasis, and shorter survival rate. Acquired PTX resistance may limit the long-term effectiveness of chemotherapy, and several mechanisms have been implicated in this process. One well-documented route in the literature is the overexpression of ATP-binding cassette (ABC) transporters, such as *ABCB1*, ABCC family members, and *ABCG2*, which pump chemotherapeutics out of the cell and maintain intracellular levels low [35]. ABCB1 contributes to the development of acquired PTX resistance, and silencing ABCB1 enhances PTX efficacy in resistant breast cancer cells [36]. In our study, PTX altered resistance-gene expression across all tested subtypes, but the patterns differed by subtype. Increased expression of *ABCB1*, *ABCC1*, *ABCC2*, *ABCC5*, and *ABCG2* genes was observed in luminal A, luminal B, and HER2-enriched BC cells; this may indicate increased drug capacity, leading to a decrease in the cytotoxic effect of PTX. MCF-7 cells also upregulated DNA repair genes (*TOP1*, *TOP2B*, *XPC*, *ERCC3*, *CLPTM1L*), suggesting that the DNA damage response is activated as part of an adaptive resistance program. *TP53* and *BAX* increased in the same cells, indicating that pro-apoptotic signaling was running alongside repair. However, HER2+ SK-BR-3 cells exhibited the opposite effect. *EPHX1*, *HIF1A*, *UGCG*, *XPA*, *CLPTM1L*, and *FOS* all showed marked decreases, suggesting that PTX partially suppressed DNA repair and survival pathways in this line. TNBC cells went in the same direction, with a long list of resistance genes dropping (*BCL2*, *ABCC1*, *ABCC2*, *TOP2A*, *TOP2B*, *MVP*, *TOP1*, *ATM*, *MYC*, *HIF1A*, *MSH2*, *SOD1*, *UGCG*, *XPA*). That pattern fits a more PTX-sensitive state and less resistance capacity. Even though TNBC is aggressive by every other measure, PTX seems to shut down several of its resistance pathways. Taken together, these subtype-specific changes indicate that PTX induced subtype-dependent expression changes in resistance-associated genes; however, these transcriptional patterns do not establish functional PTX resistance or sensitivity and require validation in resistant models and functional assays.

The KEGG enrichment analysis indicated that PTX-treated BC cell lines shared a group of core cancer-related pathways, including central carbon metabolism, fatty acid degradation, PPAR signaling, p53 signaling, HIF-1 signaling, PI3K/Akt signaling, MAPK signaling, Rap1 signaling, and Ras signaling. However, these pathway names are not expected to appear directly in the gene–metabolite interaction networks, because Figure 10, Figure 11, Figure 12 and Figure 13 represent database-derived gene–metabolite associations rather than KEGG pathway-level enrichment outputs. Instead, the metabolic components of these core pathways are represented as interconnected metabolite–gene modules. In this context, the revised gene–metabolite networks clarify how the shared KEGG-enriched cancer pathways are reflected at the metabolite-associated gene level. Across the four breast cancer cell lines, common modules related to glycolysis/pentose phosphate metabolism, TCA/anaplerotic metabolism, lipid/fatty acid metabolism, and nucleotide/energy metabolism were observed. Nevertheless, the organization of these modules differed among subtypes. MCF-7 cells showed a network centered on glucose-6-phosphate, *G6PD*, NADP, ATP/ADP, glycerol, and palmitoyl-CoA, suggesting a luminal A-associated response involving redox metabolism and lipid remodeling. SK-BR-3 cells showed stronger representation of TCA/anaplerotic metabolites, including succinic acid, oxaloacetic acid, oxoglutaric acid, and L-glutamic acid, consistent with a HER2-enriched metabolic response. BT-474 cells displayed combined lipid, glycolytic/lactate, and anaplerotic features involving *ACADL*, *LDHA/LDHAL6A*, *GOT1*, palmitoyl-CoA, oxoglutaric acid, and oxaloacetic acid. In contrast, MDA-MB-231 cells exhibited a broader and more interconnected network involving ATP, ADP, GTP, NADP, glucose-6-phosphate, palmitoyl-CoA, succinyl-CoA, *LDHA*, *OGDH*, *GOT1*, *ACAT1*, *NME2*, and *UCKL1*, supporting a more metabolically plastic and stress-adaptive phenotype in the triple-negative model. Thus, the revised analysis indicates that the core cancer pathways identified by KEGG enrichment are not absent from the gene–metabolite networks; rather, they are represented through subtype-specific metabolite–gene interaction modules. These findings strengthen the interpretation that PTX induces both shared and subtype-specific metabolic adaptations in BC cells.

Despite providing a parallel comparison of PTX responses across molecularly distinct BC cell models, this study has several limitations. First, the findings are based on in vitro cell line models and may not fully reflect the complexity of in vivo tumors, including tumor microenvironmental, immune, stromal, and pharmacokinetic factors. Second, although MCF-7, BT-474, SK-BR-3, and MDA-MB-231 cells were selected to represent different BC phenotypes, each subtype was represented by a single cell line. Therefore, the subtype-associated differences observed here should be validated in additional cell lines, patient-derived models, organoids, in vivo systems, and clinically characterized tumor samples. Third, cell viability was mainly assessed using the MTT assay, which reflects metabolic activity rather than directly measuring cell number or long-term clonogenic survival. Therefore, the antiproliferative effects of PTX should be confirmed using complementary functional assays. Fourth, targeted RT-qPCR panels were used instead of whole-transcriptome profiling; therefore, some genes and regulatory networks, including *CASP3* mRNA, may not have been captured. Finally, the gene–metabolite interaction networks were based on database-derived associations and should be interpreted as predictive, hypothesis-generating models rather than direct evidence of altered metabolite abundance or metabolic flux. Further metabolomic, functional, resistance-model, and clinically relevant studies are needed to validate these findings and clarify the mechanisms underlying PTX response and resistance.

## 4. Materials and Methods

### 4.1. Cell Lines, Reagents, and Culture Conditions

Human BC cell lines MCF-7 (ATCC HTB-22), BT-474 (ATCC HTB-20), SK-BR-3 (ATCC HTB-30), and MDA-MB-231 (ATCC HTB-26), together with the non-tumorigenic breast epithelial line hTERT-HME1 (ATCC CRL-4010), were obtained from the American Type Culture Collection (ATCC, Manassas, VA, USA). The cell lines were selected to represent distinct breast cancer phenotypes based on hormone receptor and HER2 status. MCF-7 cells were used as an HR-positive/HER2-negative luminal A model; BT-474 cells as an HR-positive/HER2-positive luminal B model; SK-BR-3 cells as an HR-negative/HER2-overexpressing HER2-enriched model; and MDA-MB-231 cells as a triple-negative BC model. This panel was intended to enable an exploratory comparison of PTX responses across biologically distinct breast cancer models rather than to provide comprehensive coverage of all molecular subtypes.

Paclitaxel (PTX; Merck, Darmstadt, Germany) was dissolved in DMSO to prepare a 50 mM stock solution and stored at −20 °C until use; the final DMSO concentration in all assays was ≤0.1%. General cell culture conditions, MTT cytotoxicity assays, total RNA isolation, cDNA synthesis, SYBR Green-based RT-qPCR with the 2^−ΔΔCT^ method, FITC Annexin V/PI flow cytometry apoptosis assays, and PI flow cytometry cell cycle analyses were performed as previously described by our group [37], with the PTX- and cell line-specific modifications outlined below.

Primary antibodies against caspase-3 (Cat. No. A2156), Bax (Cat. No. A11931), Bcl-2 (Cat. No. A19693), Bcl-xL (Cat. No. A0209), phospho-CDK2 (Cat. No. AP0325), CDK4 (Cat. No. A11136), Cyclin D1 (Cat. No. A19038), CDK2 (Cat. No. A0294), phospho-CDK4 (Cat. No. AP0593), and CDKN1A/p21CIP1 (Cat. No. A19094) were purchased from ABclonal Technology (Wuhan, China).

### 4.2. PTX Cytotoxicity and Selectivity

Cells were seeded at 6 × 10^3^ cells/100 µL in 96-well plates, allowed to attach, and exposed to nine PTX concentrations (1000, 800, 400, 200, 50, 20, 10, 5, and 2 nM), with points evaluated for 24, 48, and 72 h. Each concentration was tested in technical triplicate in three independent experiments. IC_50_ values for each cell line and time point were calculated from the resulting concentration–response curves by nonlinear regression using GraphPad Prism 10.0.0. To evaluate selectivity, the same concentration series was used for hTERT-HME1 cells, and the selectivity index (SI) was calculated as SI = IC_50_ (hTERT-HME1)/IC_50_ (cancer line) at 48 h [37,38]. The 48-h IC_50_ values were selected for subsequent experiments because this intermediate exposure period yielded a clear, reproducible cytotoxic response while preserving sufficient viable cells for downstream analyses. In comparison, 24 h may be insufficient for the full development of PTX-induced mitotic arrest and subsequent cell death, whereas 72 h may lead to more extensive cell loss and secondary effects associated with prolonged treatment. The use of a 48-h exposure period is also consistent with previous in vitro PTX studies in breast cancer cell lines [38].

### 4.3. RT-qPCR Gene Expression Profiling

For RT-qPCR experiments, cells were seeded at 2.5 × 10^5^ cells/well in 6-well plates, allowed to attach for 24 h, then exposed to the cell line-specific 48-h IC_50_ concentration of PTX (MCF-7: 12.60 nM; BT-474: 5.09 nM; SK-BR-3: 16.09 nM; MDA-MB-231: 36.66 nM) for an additional 48 h. Total RNA was extracted using a QIAGEN RNA İsolation Kit (Qiagen, Düsseldorf, Germany) and reverse-transcribed into cDNA using a Wonder RT cDNA Synthesis kit (Euroclone, Pero, Italy) according to the manufacturer’s instructions. Cancer pathway, energy-metabolism, and drug-resistance primer panels were used (Merck, Darmstadt, Germany; full panels in Supplementary Tables S1 and S2), and ACTB and GAPDH were used as reference genes. Reactions were performed using SYBR Green Master Mix (Thermo Fisher Scientific, Waltham, MA, USA), with an initial denaturation at 95 °C for 5 min, followed by 45 cycles of 95 °C for 15 s and 60 °C for 30 s. All RT-qPCR reactions were performed in three biological replicates; fold-change values were reported descriptively. Genes with ≥2-fold upregulation or ≤0.5-fold downregulation were retained for KEGG pathway enrichment analysis using ShinyGO 0.82 [37,39,40,41,42]. Enriched pathways were ranked by fold enrichment, and significance was adjusted for multiple testing using the Benjamini–Hochberg false discovery rate (FDR) correction implemented in ShinyGO0.82.

### 4.4. Apoptosis and Cell-Cycle Assay

For apoptosis and cell-cycle experiments, cells were seeded at 3 × 10^5^ cells/well in 6-well plates and were treated with PTX at the cell line-specific 48-h IC_50_ concentration listed in Section 4.3 for 48 h. Apoptosis was measured with the Annexin V/FITC Apoptosis Detection Kit (Elabscience, Houston, TX, USA) and PI co-staining. Cell-cycle distribution was measured with the Cell Cycle Analysis Kit (Elabscience, Houston, TX, USA). Both assays were read on a Beckman Coulter Cytoflex flow cytometer (Indianapolis, IN, USA) [37].

### 4.5. Metabolite-Associated Gene Network Analysis

Alterations in gene expression profiles were identified, and their associated metabolites were explored using the Enrichr platform and the Metabolomics Workbench Metabolites 2022 repository [43,44]. Gene–metabolite interaction networks were subsequently generated using MetaboAnalyst 6.0 [45]. To clarify the relationship between KEGG pathway enrichment and gene–metabolite network topology, the generated networks were further annotated according to major biochemical modules, including glycolysis/pentose phosphate metabolism, TCA/anaplerotic metabolism, lipid/fatty acid metabolism, and nucleotide/energy metabolism. Cluster assignment was based on the known biochemical roles of the connected metabolites and genes. It was used only for interpretive visualization of the networks, not as a separate statistical enrichment test.

### 4.6. Western Blot Analysis

The expression levels of apoptosis- and cell cycle-related proteins were evaluated in control and PTX-treated MCF-7, BT-474, SK-BR-3, and MDA-MB-231 BC cells by Western blotting, with modifications to the method described by Yucel et al. [46]. Cells were seeded at a density of 4 × 10^5^ cells/well and treated with the corresponding cell line-specific 48-h IC_50_ concentration of PTX for 48 h. Following treatment, the cells were collected by centrifugation and washed with ice-cold phosphate-buffered saline (PBS). Cell pellets were lysed in RIPA buffer including protease and phosphatase inhibitor cocktails. The cell lysates were centrifuged at 12,000 rpm for 5 min at 4 °C, and the supernatants were collected as total protein lysates. Protein amounts were determined using the Bradford assay. Equivalent quantities of protein samples were separated by the SDS-PAGE method and then transferred onto PVDF membranes. The membranes were blocked with 3% nonfat dry milk and kept overnight at 4 °C with primary antibodies against caspase-3, Bax, Bcl-2, Bcl-xL, cyclin D1, CDK2, phospho-CDK2, CDK4, phospho-CDK4, and CDKN1A/p21CIP1. β-Actin was used as the loading control. Following primary antibody incubation, the membranes were washed with Tris-buffered saline containing Tween-20 and incubated with the appropriate horseradish peroxidase-conjugated secondary antibodies for 2 h at room temperature. After further washing, protein bands were visualized using an enhanced chemiluminescence substrate, and images were captured using an Azure C300 gel imaging system. The expression levels of caspase-3, Bax, Bcl-2, Bcl-xL, cyclin D1, CDK2, CDK4, and CDKN1A/p21CIP1 were normalized to β-actin, whereas phospho-CDK2 and phospho-CDK4 levels were evaluated relative to their corresponding total protein levels [46].

### 4.7. Statistical Analysis

Each experiment was performed as three independent biological experiments with three technical replicates. Results are reported as the mean ± SD. Two-group comparisons used the unpaired two-tailed Student’s *t*-test, and multi-group comparisons used one-way ANOVA with Tukey’s post hoc test. The significance threshold was selected to be 0.05. Analyses were performed in GraphPad Prism 8.0.2.

## Figures and Tables

**Table 1:** IC_50_ values and SI values of PTX in BC cell lines.

Cell Line	IC_50_ (nM)	SI (IC_50_ hTERT-HME1/IC_50_ Tumor Cell)
MCF-7	12.60 ± 0.90	0.47
BT-474	5.09 ± 0.48	1.17
SK-BR-3	16.09 ± 1.21	0.37
MDA-MB-231	36.66 ± 2.31	0.16
hTERT-HME1	5.97 ± 0.4	N/A

**Table 2:** Quantitative analysis of apoptotic cell populations in BC cell lines following 48 h paclitaxel (PTX) treatment. Q1, necrotic cells; Q2, late apoptotic/secondary necrotic cells; Q3, viable cells; Q4, early apoptotic cells. Total apoptosis was calculated as the sum of the early and late apoptotic populations (Q2 + Q4). Statistical comparisons between treated and corresponding untreated control cells were performed using a one-sample binomial test. A *p*-value < 0.05 was considered statistically significant.

Cell Line/Treatment	Q1 (Necrotic, %)	Q2 (Late Apoptotic, %)	Q3 (Viable, %)	Q4 (Early Apoptotic, %)	Total Apoptosis (Q2 + Q4, %)
MCF-7	18.47	19.78	58.39	3.36	23.14
BT-474	0.31	18.97	65.53	15.19	34.16
SK-BR-3	10.24	8.90	78.62	2.23	11.13
MDA-MB-231	0.30	7.23	86.64	5.83	13.06
Comparisons with control (*p*-values)					
Comparison	Early apoptosis	Late apoptosis	Total apoptosis (Q2 + Q4)		Necrotic
MCF-7 vs. Control	*p* = 0.0001	*p* = 0.0002	*p* = 0.0001		*p* = 0.0001
BT-474 vs. Control	*p* = 0.0001	*p* = 0.0001	*p* = 0.0001		*p* = 0.0006
SK-BR-3 vs. Control	*p* = 0.0001	*p* = 0.0001	*p* = 0.0001		*p* = 0.0001
MDA-MB-231 vs. Control	*p* = 0.0001	*p* = 0.0001	*p* = 0.0001		*p* = 0.0005

## Data Availability

The original contributions presented in this study are included in the article/Supplementary Material. Further inquiries can be directed to the corresponding author.

## Supplementary Materials

The following supporting information can be downloaded at: https://www.mdpi.com/article/10.3390/molecules31142431/s1.

## Abbreviations

The following abbreviations are used in this manuscript:

PTX	Paclitaxel
BC	Breast Cancer
TNBC	Triple Negative Breast Cancer
EMT	Epithelial–Mesenchymal Transition
HER2+	Human Epidermal Growth Factor Receptor 2 Positive
HSP	Heat Shock Protein
DDR	DNA Damage Response
HIFs	Hypoxia-inducible factors
CDKs	Cyclin-Dependent Kinases
