# Supplementary material for: Molecular Mechanisms and Molecular Subtype-Specific Responses to Paclitaxel in Breast Cancer Cells
Source: Molecules. 2026 Jul 11;31(14):2431. doi: 10.3390/molecules31142431 (PMC13414311; doi:10.3390/molecules31142431)
Supplement: Supplementary file 1 [file molecules-31-02431-s001.zip › molecules-4375601-supplementary.pdf]

Supplementary File

Supplementary Table S1: List of primers used for RT-qPCR analysis in this study.

ACAD1	CASP7	ERCC5	IGFBP5	PDK	SNAI2
ACADL	CASP9	ETS2	IGFBP7	PFKFB4	SNAI3
ACLY	CCL2	FASLG	KDR	PFKL	SOD1
ACO2	CCND2	FGF2	KRT14	PGAM1	SOX10
ACSL1	CCND3	FH	LDHA	PGF	STMN1
ACSL3	CDC20	FLT1	LDHAL6A	PGM2	TBX2
ACSL4	CDC37	FOXC2	LIG4	PHGD	TEK
ACSS3	CDH2	G6PD	LPL	PINX1	TEP1
ADCY3	CDK4	GADD45G	MAP2K1	POLB	TERF1
ADM	CFLAR	GLDC	MAP2K3	PPP1R15A	TERF2IP
AMPD3	COX5A	GNPNAT1	MAPK14	PRPS1	TINF2
ANGPT1	CPT1C	GOT1	MAT2A	PSPH	TK1
ANGPT2	CPT2	GPD2	MCM2	SCD	TKTL1
APAF1	DDB2	GSC	ME1	SDHA	TNKS
ARNT	DDIT3	GUSB	MGLL	SERPINB2	TNKS2
ATP5A1	DHFR	HK2	MKI67	SERPINF1	TP53
AURKA	DKC1	HMGCS1	MTR	SKP2	TPI1
BCAT1	DNMT1	HMOX1	NME2	SLC1A5	TRAP1
BCL2L11	DSP	HSP90AB1	NOL3	SLC2A1	UCKL1
BIRC3	E2F4	HSP90AB4P	OCLN	SLC2A2	UQCRFS1
BMI1	ENO1	HSPB1	OGDH	SLC2A7	VEGFC
CA9	EPO	IDH1	PAFAH1B2	SLC7A11	WEE1
CASP2	ERCC3	IGFBP3	PDE4D	SNAI1	XIAP

Supplementary Table S2: List of primers associated with drug resistance.

ABCB1	APC	BRCA1	FOS	SOD1	UGCG
ABCC1	ATM	BRCA2	GSK3A	SULT1E1	XPA
ABCC2	BCL2	CLPTM1L	HIF1A	TOP1	XPC
ABCC3	BCL2L1	CMYC	MSH2	TOP2A	
ABCC5	BLMH	EPHX1	MVP	TOP2B	
ABCG2	BOX	ERCC3	RB1	TPMT	

Supplementary Table S3: RT-qPCR-based analysis of gene expression for the primers listed in Supplementary Table 1 in MCF-7, SK-BR-3, BT-474, and MDA-MB-231 cell lines. Relative expression levels were calculated using the $2^{-\Delta\Delta Ct}$ method and normalized to the control group (set as 1). All experiments were performed in triplicate. Statistical analysis was conducted using two-way ANOVA with comparisons made against the control group, followed by Dunnett's multiple comparisons test. Data are presented as mean values, and statistical significance is indicated as follows: *p < 0.05, **p < 0.01, ***p < 0.001, and **p < 0.0001.

Gene	MCF-7	SK-BR-3	BT-474	MDA-MB-231	Gene	MCF-7	SK-BR-3	BT474	MDA-MB-231
ACAD1	-3.24 ± 0.30****	8.00 ± 1.77****	-21.94 ± 2.91****	-5.04 ± 0.92****	***IGFBP5***	15.49 ± 2.40****	7.68 ± 0.98****	1.88 ± 0.50	6.89 ± 1.71****
ACADL	3.73 ± 0.60***	5.66 ± 1.16****	-4.06 ± 1.71****	3.65 ± 0.89***	***IGFBP7***	12.13 ± 1.78****	-2.06 ± 0.30****	1.59 ± 0.40	-3.32 ± 0.52****
ACLY	-3.22 ± 0.40****	-2.55 ± 0.32****	1.78 ± 0.46	1.82 ± 0.33	***KDR***	6.64 ± 1.12****	11.19 ± 1.67****	7.52 ± 2.10****	6.64 ± 1.50****
ACO2	2.38 ± 0.27	3.44 ± 0.74***	-9.40 ± 3.69****	0.18 ± 1.25	***KRT14***	6.96 ± 1.18****	1.44 ± 0.22	16.01 ± 1.96****	-1.92 ± 0.60****
ACSL1	2.99 ± 0.25**	7.80 ± 1.52****	2.03 ± 0.75	-2.44 ± 0.77****	***LDHA***	-11.06 ± 1.19****	-1.58 ± 0.19***	0.08 ± 1.29	-1.28 ± 0.12**
ACSL3	1.83 ± 0. 17	3.20 ± 0.70**	-2.14 ± 0.80****	-2.30 ± 0.44****	***LDHAL6A***	2.66 ± 0.45*	-1.50 ± 0.31***	-4.93 ± 2.04****	1.74 ± 0.41
ACSL4	2.14 ± 0.25	3.13 ± 0.44**	9.89 ± 2.59****	-1.49 ± 0.29***	***LIG4***	0.01 ± 1.17	1.91 ± 0.27	3.97 ± 1.09****	1.86 ± 0.46
ACSS3	-2.65 ± 0.32****	-3.01 ± 0.62****	3.42 ± 1.32***	-2.59 ± 0.50****	***LPL***	5.02 ± 0.72****	16.48 ± 2.35****	1.93 ± 0.53	3.45 ± 0.85***
ADCY3	-13.14 ± 1.84****	1.56 ± 0.33	-5.84 ± 2.33****	-1.39 ± 0.32***	***MAP2K1***	-3.94 ± 0.51****	2.97 ± 0.38**	8.35 ± 3.10****	1.53 ± 0.26
ADM	6.38 ± 0.85****	8.91 ± 1.25****	13.83 ± 0.22****	1.40 ± 0.27	***MAP2K3***	-13.52 ± 1.5****	2.32 ± 0.38	-0.26 ± 1.33	1.66 ± 0.27
AMPD3	2.56 ± 0.40	2.43 ± 0.51	-1.92 ± 0.76****	1.72 ± 0.35	***MAPK14***	-2.12 ± 0.28****	6.52 ± 0.82****	-6.42 ± 1.70****	0.04 ± 1.10
ANGPT1	-6.42 ± 1.16****	-12.04 ± 1.81****	3.34 ± 0.90**	4.47 ± 1.21****	***MAT2A***	1.11 ± 0.10	-1.33 ± 0.29**	-1.77 ± 0.66****	-1.68 ± 0.30***
ANGPT2	-4.89 ± 0.86****	5.73 ± 0.84****	-2.59 ± 0.73****	5.06 ± 1.51****	***MCM2***	-2.29 ± 0.30****	1.62 ± 0.20	-2.67 ± 0.70****	0.02 ± 1.04

APAF1	-2.75 ± 0.46****	7.52 ± 1.03****	1.77 ± 0.48	1.58 ± 0.41	***ME1***	-2.74 ± 0.36****	3.34 ± 0.72**	-6.92 ± 2.66****	-2.87 ± 1.61****
ARNT	-4.57 ± 0.68****	-1.37 ± 0.18**	0.17 ± 1.32	3.87 ± 0.74****	***MGLL***	11.66 ± 1.35****	2.72 ± 0.59*	-15.52 ± 2.92****	4.89 ± 0.76****
ATP5A1	-7.37 ± 0.83****	2.47 ± 0.29	-3.95 ±1.02****	16.80 ± 0.29****	***MKI67***	-1.28 ± 0.13**	2.57 ± 0.31	0.25 ± 1.32	2.17 ± 0.28
AURKA	-1.48 ± 0.17***	1.60 ± 0.19	-5.59 ± 1.43****	2.16 ± 0.31	***MTR***	-1.40 ± 0.16***	1.83 ± 0.39	-1.87 ± 0.75****	-1.50 ± 0.34***
BCAT1	12.18 ± 1.29****	-1.28 ± 0.28**	1.98 ± 0.75	4.34 ± 0.72****	***NME2***	-1.99 ± 0.30****	-1.29 ± 0.26**	2.12 ± 0.78	-4.05 ± 0.96****
BCL2L11	3.18 ± 0.42**	2.71 ± 0.35*	-3.59 ± 0.98****	2.32 ± 0.58	***NOL3***	1.77 ± 0.33	1.44 ± 0.22	-3.81 ± 1.13****	-2.13 ± 0.83****
BIRC3	-3.60 ± 0.58****	-2.97 ± 0.40****	7.66 ±1.97****	2.06 ± 0.35	***OCLN***	-1.44 ± 0.18***	1.43 ± 0.18	-3.48 ± 0.91****	-2.44 ± 0.46****
BMI1	1.20 ± 0.14	2.21 ± 0.28	-1.38 ± 0.35***	1.25 ± 0.20	***OGDH***	2.84 ± 0.35*	0.14 ± 1.25	-2.24 ± 0.86****	-1.65 ± 0.34***
CA9	-10.56 ± 1.02****	13.02 ± 2.06****	2.38 ± 0.69	9.00 ± 0.22****	***PAFAH1B2***	-2.19 ± 0.28****	2.22 ± 0.48	0.02 ± 1.48	1.30 ± 0.24
CASP2	7.53 ± 1.15****	1.55 ± 0.22	2.29 ± 0.63	-3.39 ± 0.99****	***PDE4D***	3.42 ± 0.58***	-1.32 ± 0.27**	2.18 ± 0.31	-1.68 ± 0.34***
CASP7	-7.99 ± 1.15****	2.51 ± 0.33	1.89 ± 0.50	1.80 ± 0.36	***PDK***	1.89 ± 0.34	0.22 ± 1.26	-8.48 ± 3.39****	-2.15 ± 0.83****
CASP9	-6.23 ± 1.02****	1.65 ± 0.23	3.38 ± 0.90***	-1.41 ± 0.32***	***PFKFB4***	2.90 ± 0.34*	1.47 ± 0.30	2.49 ± 0.99	3.38 ± 0.68***
CCL2	-1.86 ± 0.34****	-2.39 ± 0.35****	10.75 ± 2.93****	8.76 ± 1.69****	***PFKL***	-1.45 ± 0.20***	3.20 ± 0.42**	-1.68 ± 0.45***	0.36 ± 1.36
CCND2	14.45 ± 2.68****	-1.53 ± 0.25***	10.00 ± 3.80****	1.37 ± 0.23	***PGAM1***	2.55 ± 0.22	2.25 ± 0.49	-1.65 ± 0.60***	-11.17 ± 2.33****
CCND3	2.03 ± 0.27	1.16 ± 0.15	0.05 ± 1.31	0.24 ± 1.24	***PGF***	4.62 ± 0.85****	6.15 ± 0.94****	9.57 ± 1.72****	1.67 ± 0.53
CDC20	-1.45 ± 0.19***	2.56 ± 0.32	-1.75 ± 0.46****	1.44 ± 0.24	***PGM2***	1.15 ± 0.13	2.60 ± 0.55*	1.64 ± 0.63	-3.13 ± 1.78****
CDC37	-1.27 ± 0.14**	7.33 ± 0.92****	2.00 ± 0.51	-1.25 ± 0.23**	***PHGD***	1.14 ± 0.12	2.36 ± 0.50	-6.76 ± 2.69****	-9.67 ± 1.84****
CDH2	8.71 ± 1.29****	4.38 ± 0.66****	-16.94 ± 0.91****	-6.99 ± 0.24****	***PINX1***	7.18 ± 1.28****	-18.53 ± 2.47****	-9.52 ± 2.46****	-2.42 ± 0.43****

CDK4	14.11 ± 1.45****	10.71 ±1.29****	-1.66 ± 0.43***	0.06 ± 1.17	***POLB***	-13.64 ± 1.92****	2.13 ± 0.27	-5.94 ± 1.59****	-2.35 ± 0.54****
CFLAR	2.66 ± 0.40*	3.26 ± 0.44**	-1.71 ± 0.46***	0.01 ± 1.27	***PPP1R15A***	-1.41 ± 0.26***	14.47 ± 2.03****	14.21 ± 1.01****	1.71 ± 0.45
COX5A	-2.95 ± 0.35****	-7.99 ± 1.00****	-9.54 ± 2.44****	5.75 ± 1.43****	***PRPS1***	-1.31 ± 0.16**	4.29 ± 0.92****	-3.74 ± 1.47****	2.66 ± 0.55*
CPT1C	-1.82 ± 0.23****	16.14 ± 3.32****	7.15 ± 0.58****	-1.39 ± 0.35****	***PSPH***	1.17 ± 0.15	2.37 ± 0.51	-3.42 ± 1.34****	-2.52 ± 1.10****
CPT2	-3.00 ± 0.44****	1.33 ± 0.17	-2.46 ± 0.66****	0.24 ± 1.27	***SCD***	1.58 ± 0.12	2.04 ± 0.45	-7.30 ± 2.68****	1.98 ± 0.33
DDB2	3.90 ± 0.46****	11.06 ± 1.45****	-5.76 ± 1.57****	1.73 ± 0.40	***SDHA***	-2.36 ±0.27****	-5.07 ± 1.08****	-5.56 ± 2.38****	-1.90 ± 0.52****
DDIT3	2.61 ± 0.31*	2.46 ± 0.32	2.99 ± 0.76**	1.34 ± 0.18	***SERPINB2***	5.40 ± 0.80****	5.59 ± 0.80****	10.46 ± 0.49****	9.31 ± 1.81****
DHFR	-6.51 ± 0.62****	2.54 ± 0.56	-2.10 ± 0.79****	2.51 ± 0.46	***SERPINF1***	2.24 ± 0.36	13.28 ± 1.91****	-2.05 ± 0.58****	3.33 ± 1.00**
DKC1	2.78 ± 0.30*	7.98 ± 1.05****	-0.20 ± 1.34	6.08 ± 1.15****	***SKP2***	-13.44 ± 1.97****	5.52 ± 0.69****	-4.48 ± 1.19****	0.15 ± 1.26
DNMT1	-1.15 ± 0.11**	1.97 ± 0.43	-9.73 ± 3.72****	0.14 ± 1.21	***SLC1A5***	-9.77 ± 1.58****	2.06 ± 0.44	1.70 ± 0.64	-1.57 ± 0.32***
DSP	-9.25 ± 1.24****	6.19 ± 0.75****	-10.54 ± 2.75****	1.38 ± 0.24	***SLC2A1***	2.14 ± 0.23	2.77 ± 0.60*	2.76 ± 1.03*	-1.79 ± 0.35****
E2F4	1.62 ± 0.21	7.10 ± 0.93****	0.07 ± 1.31	0.19 ± 1.24	***SLC2A2***	-2.98 ± 0.49****	-1.36 ± 0.28**	-14.20 ± 1.25****	13.00 ± 3.05****
ENO1	1.82 ± 0.14	-2.32 ± 0.51****	0.35 ± 1.49	1.29 ± 0.20	***SLC2A7***	1.31 ± 0.15	-2.27 ± 0.46****	-4.02 ± 1.72****	1.51 ± 0.29
EPO	8.99 ± 1.36****	12.03 ± 1.75****	-2.63 ± 0.76****	-1.63 ± 0.61***	***SLC7A11***	-2.05 ± 0.28****	-1.70 ± 0.36***	-6.59 ± 2.73****	-1.60 ± 0.30***
ERCC3	7.19 ± 0.73****	2.69 ± 0.35*	0.17 ± 1.32	2.19 ± 0.40	***SNAI1***	3.51 ± 0.62***	10.83 ± 1.59****	6.91 ± 1.2****	13.09 ± 0.41****
ERCC5	11.48 ± 1.44****	0.14 ± 1.15	-1.38 ± 0.37***	1.48 ± 0.33	***SNAI2***	13.09 ± 1.79****	4.55 ± 0.68****	4.15 ± 1.13****	1.58 ± 0.35

ETS2	5.05 ± 0.76****	1.68 ± 0.23	10.65 ± 2.06****	0.05 ± 1.11	***SNAI3***	5.99 ± 1.06****	-11.61 ± 1.93****	-17.46 ± 0.57****	-1.89 ± 0.73****
FASLG	1.56 ± 0.29	1.30 ± 0.18	-3.57 ± 1.06****	-5.13 ± 1.89****	***SOD1***	-3.89 ± 0.36****	1.72 ± 0.20	-12.51 ± 3.19****	2.65 ± 0.29*
FGF2	-11.11 ± 1.91****	1.45 ± 0.23	2.08 ± 0.60	-5.77 ± 0.66****	***SOX10***	3.72 ± 0.55***	9.10 ± 1.35****	-11.77 ± 0.59****	12.46 ± 1.72****
FH	2.24 ± 0.24	2.95 ± 0.65**	-10.14 ± 3.79****	1.76 ± 0.34	***STMN1***	-3.68 ± 0.68****	4.03 ± 0.58****	0.17 ± 1.34	3.11 ± 0.85**
FLT1	-5.14 ± 0.91****	6.89 ± 1.03****	-8.63 ± 2.47****	-13.76 ± 2.41****	***TBX2***	19.88 ± 2.58****	16.65 ± 2.32****	-8.47 ± 2.33****	1.59 ± 0.53
FOXC2	14.94 ± 2.45****	-1.56 ± 0.25***	-1.74 ± 0.52****	-6.41 ± 1.08****	***TEK***	-2.50 ± 0.44	7.19 ± 1.06	-2.09± 0.59	4.94 ± 1.53
G6PD	-1.86 ± 0.22****	1.77 ± 0.38	-14.50 ± 0.03****	-5.27 ± 1.34****	***TEP1***	-8.33 ± 1.06***	0.13 ± 1.15	0.28 ± 1.34	1.47 ± 0.34
GADD45G	4.87 ± 0.89****	8.93 ± 1.30****	-10.81 ± 3.05****	2.62 ± 0.90*	***TERF1***	2.87 ± 0.29*	-1.30 ± 0.16**	-7.09 ± 1.86****	-10.58 ± 0.70****
GLDC	-1.66 ± 0.25***	-1.77 ± 0.36****	-10.60 ± 2.57****	-2.65 ± 1.09****	***TERF2IP***	-1.24 ± 0.16**	3.62 ± 0.48***	0.29 ± 1.35	0.04 ± 1.26
GNPNAT1	1.48 ± 0.16	3.09 ± 0.67**	-4.08 ± 1.58	1.38 ± 0.27****	***TINF2***	-1.36 ± 0.18**	7.86 ± 0.99****	-5.40 ± 1.43****	1.47 ± 0.26
GOT1	0.13 ± 1.15	2.39 ± 0.51	-2.24 ± 0.86****	-2.41 ± 0.97****	***TK1***	-2.62 ± 0.34****	0.13 ± 1.25	-1.67 ± 0.40***	1.55 ± 0.33
GPD2	-1.85 ± 0.25****	0.06 ± 1.14	-1.53 ± 0.41***	2.35 ± 0.43	***TKTL1***	-9.27 ± 1.47****	-3.25 ± 0.66****	-20.01 ± 0.17****	-5.10 ± 1.20****
GSC	1.76 ± 0.31	3.85 ± 0.56****	6.48 ± 1.80****	-7.90 ± 2.57****	***TNKS***	1.53 ± 0.20	0.13 ± 1.15	1.84 ± 0.49	1.43 ± 0.28
GUSB	3.62 ± 0.59***	5.60 ± 0.83****	15.23 ± 3.94****	-3.09 ± 0.65****	***TNKS2***	1.20 ± 0.16	8.17 ± 1.06****	-1.61 ± 0.43***	1.29 ± 0.24
HK2	1.35 ± 0.20	2.17 ± 0.46	0.43 ± 1.58	0.09 ± 1.25	***TP53***	7.76 ± 0.92****	6.65 ± 0.84****	1.48 ± 0.38	1.67 ± 0.23
HMGCS1	4.53 ± 0.49****	3.21 ± 0.71**	-14.61 ± 0.53****	5.56 ± 1.11****	***TPI1***	7.87± 0.68****	1.93 ± 0.43	-3.82 ± 1.39****	2.82 ± 0.45*
HMOX1	1.58 ± 0.24	-1.26 ± 0.18**	6.46 ± 1.78****	3.54 ± 0.73***	***TRAP1***	-1.70± 0.24***	1.91 ± 0.24	-3.85 ± 1.02****	-1.91 ± 0.40****

HSP90AB1	$3.18 \pm 0.32^{**}$	$3.61 \pm 0.41^{***}$	$-4.43 \pm 1.12^{****}$	1.07 ± 0.02	***UCKL1***	1.59 ± 0.19	2.30 ± 0.49	$-2.78 \pm 1.08^{****}$	$-2.89 \pm 1.44^{****}$
HSP90AB4P	$-2.04 \pm 0.37^{****}$	$9.04 \pm 1.33^{****}$	$-12.05 \pm 3.44^{****}$	$2.81 \pm 0.90^{*}$	***UQCRFS1***	$-12.12 \pm 1.49^{****}$	1.35 ± 0.17	$-3.70 \pm 0.96^{****}$	1.91 ± 0.33
HSPB1	$21.81 \pm 2.14^{****}$	$12.50 \pm 1.48^{****}$	$2.66 \pm 0.68^{*}$	1.97 ± 0.38	***VEGFC***	$9.43 \pm 1.40^{****}$	$4.01 \pm 0.54^{****}$	$9.41 \pm 0.70^{****}$	1.96 ± 0.32
IDH1	1.80 ± 0.16	$4.82 \pm 1.06^{****}$	0.25 ± 1.56	$-1.73 \pm 0.35^{***}$	***WEE1***	$-9.03 \pm 1.22^{****}$	$5.13 \pm 0.62^{****}$	$-5.96 \pm 1.57^{****}$	1.78 ± 0.32
IGFBP3	$3.20 \pm 0.42^{**}$	$7.94 \pm 1.09^{****}$	$14.92 \pm 1.16^{****}$	1.55 ± 0.27	***XIAP***	1.30 ± 0.19	$6.18 \pm 0.81^{****}$	0.23 ± 1.34	$-1.35 \pm 0.31^{**}$

Supplementary Table S4: KEGG pathway enrichment analysis of significantly dysregulated genes in MCF-7 cells following paclitaxel treatment, generated using ShinyGO. Genes with ≥2-fold upregulation or ≤0.5-fold downregulation were included in the analysis. The table presents enriched KEGG pathways (Pathways), false discovery rate values (Enrichment FDR), number of genes associated with each pathway (nGenes), genes contributing to pathway enrichment (Pathway Genes), and fold enrichment values (Fold Enrichment). Adjusted p-values were calculated using the Benjamini–Hochberg correction method, and statistical significance was accepted at FDR-adjusted $p < 0.05$. Accessed May 16, 2026.

**Pathways**	**Enrichment FDR**	**nGenes**	**Pathway Genes**	**Fold Enrichment**
Central carbon metabolism in cancer	7.7E-11	10	71	29.5
Fatty acid degradation	8.5E-07	6	43	29.2
P53 signaling pathway	2.5E-09	9	75	25.1
Carbon metabolism	1.3E-12	13	115	23.7
Biosynthesis of amino acids	6.2E-08	8	75	22.4
PPAR signaling	6.3E-08	8	76	22.1

HIF-1 signaling pathway	1.9E-10	11	109	21.1
Small cell lung cancer	2.7E-07	8	93	18
Apoptosis	1.8E-09	11	137	16.8
TNF signaling pathway	1.4E-06	8	119	14.1
Epstein-Barr virus infection	6.2E-09	12	202	12.4
Cellular senescence	9.6E-07	9	157	12
Cell cycle	9.6E-07	9	158	11.9
Rap1 signaling pathway	1.1E-07	11	211	10.9
MAPK signaling pathway	5.1E-10	15	300	10.5
Thermogenesis	2.8E-07	11	234	9.8
PI3K-Akt signaling pathway	1.7E-11	17	362	9.8
Human T-cell leukemia virus 1 infection	1.5E-06	10	223	9.4
Pathways in cancer	6.1E-12	21	529	8.3
Metabolic pathways	1.7E-14	37	1556	5

Supplementary Table S5: KEGG pathway enrichment analysis of significantly dysregulated genes in SK-BR-3 cells following paclitaxel treatment, generated using ShinyGO. Genes with ≥2-fold upregulation or ≤0.5-fold downregulation were included in the analysis. The table presents enriched KEGG pathways (Pathways), false discovery rate values (Enrichment FDR), number of genes associated with each pathway (nGenes), genes contributing to pathway enrichment (Pathway Genes), and fold enrichment values (Fold Enrichment). Adjusted p-values were

calculated using the Benjamini–Hochberg correction method, and statistical significance was accepted at FDR-adjusted $p < 0.05$. Accessed May 16, 2026.

**Pathways**	**Enrichment FDR**	**nGenes**	**Pathway Genes**	**Fold Enrichment**
Citrate cycle (TCA cycle)	2.7E-06	5	30	37.3
Central carbon metabolism in cancer	1.0E-12	11	71	34.6
Biosynthesis of amino acids	1.5E-12	11	75	32.8
Fatty acid degradation	5.4E-07	6	43	31.2
Carbon metabolism	4.0E-16	15	115	29.2
HIF-1 signaling pathway	4.7E-15	14	109	28.7
Fatty acid metabolism	1.3E-07	7	57	22.5
PPAR signaling	1.7E-09	9	76	26.5
P53 signaling pathway	6.0E-07	7	75	20.9
Small cell lung cancer	1.7E-07	8	93	19.2
TNF signaling pathway	7.1E-08	9	119	16.9
Apoptosis	1.4E-08	10	137	16.3
Cellular senescence	5.4E-07	9	157	12.8
MAPK signaling pathway	1.9E-10	15	300	11.2

Epstein-Barr virus infection	4.3E-07	10	202	11.1
Rap1 signaling pathway	5.4E-07	10	211	10.6
Transcriptional misregulation in cancer	2.8E-06	9	195	10.3
PI3K-Akt signaling pathway	2.1E-09	15	362	9.3
Pathways in cancer	8.4E-12	20	529	8.5
Metabolic pathways	2.0E-17	39	1556	5.6

Supplementary Table S6: KEGG pathway enrichment analysis of significantly dysregulated genes in BT-474 cells following paclitaxel treatment, generated using ShinyGO. Genes with ≥2-fold upregulation or ≤0.5-fold downregulation were included in the analysis. The table presents enriched KEGG pathways (Pathways), false discovery rate values (Enrichment FDR), number of genes associated with each pathway (nGenes), genes contributing to pathway enrichment (Pathway Genes), and fold enrichment values (Fold Enrichment). Adjusted p-values were calculated using the Benjamini–Hochberg correction method, and statistical significance was accepted at FDR-adjusted $p < 0.05$. Accessed May 16, 2026.

**Pathways**	**Enrichment FDR**	**nGenes**	**Pathway Genes**	**Fold Enrichment**
Fatty acid degradation	2.1E-08	7	43	36.1
Fatty acid metabolism	5.2E-09	8	57	31.1
Biosynthesis of amino acids	1.3E-10	10	75	29.5
Carbon metabolism	5.0E-16	15	115	28.9
PPAR signaling	2.5E-09	9	76	26.2

Central carbon metabolism in cancer	5.7E-07	7	71	21.8
Cysteine and methionine metabolism	5.0E-05	5	52	21.3
HIF-1 signaling pathway	2.5E-09	10	109	20.3
P53 signaling pathway	1.9E-05	6	75	17.7
Apoptosis	1.7E-08	10	137	16.2
FoxO signaling pathway	3.5E-05	7	132	11.7
Rap1 signaling pathway	6.7E-08	11	211	11.5
Thermogenesis	1.8E-07	11	234	10.4
MAPK signaling pathway	2.7E-09	14	300	10.3
Ras signaling pathway	1.9E-07	11	236	10.3
PI3K-Akt signaling pathway	3.9E-10	16	362	9.9
Transcriptional misregulation in cancer	4.2E-05	8	195	9.1
Epstein-Barr virus infection	5.0E-05	8	202	8.8
Pathways in cancer	1.7E-10	19	529	8
Metabolic pathways	4.1E-19	41	1556	5.8

Supplementary Table S7: KEGG pathway enrichment analysis of significantly dysregulated genes in MDA-MB-231 cells following paclitaxel treatment, generated using ShinyGO. Genes with ≥2-fold upregulation or ≤0.5-fold downregulation were included in the analysis.

The table presents enriched KEGG pathways (Pathways), false discovery rate values (Enrichment FDR), number of genes associated with each pathway (nGenes), genes contributing to pathway enrichment (Pathway Genes), and fold enrichment values (Fold Enrichment). Adjusted p-values were calculated using the Benjamini–Hochberg correction method, and statistical significance was accepted at FDR-adjusted $p < 0.05$. Accessed May 16, 2026.

**Pathways**	**Enrichment FDR**	**nGenes**	**Pathway Genes**	**Fold Enrichment**
Fatty acid degradation	2.3E-07	6	43	39.1
Biosynthesis of amino acids	9.8E-12	10	75	37.4
Ferroptosis	6.0E-06	5	41	34.2
2-Oxocarboxylic acid metabolism	7.5E-05	4	33	34
Carbon metabolism	2.2E-14	13	115	31.7
Central carbon metabolism in cancer	8.6E-09	8	71	31.6
Fatty acid metabolism	1.1E-06	6	57	29.5
PPAR signaling	1.1E-08	8	76	29.5
One carbon pool by folate	1.2E-04	4	38	29.5
Cysteine and methionine metabolism	1.9E-05	5	52	26.8
HIF-1 signaling pathway	8.6E-09	9	109	23.1
Adipocytokine signaling pathway	7.2E-05	5	70	20
Platinum drug resistance	9.0E-05	5	75	18.8

Apoptosis	4.7E-08	9	137	18.4
Thermogenesis	2.9E-05	8	234	9.6
MAPK signaling pathway	2.2E-06	10	300	9.3
Rap1 signaling pathway	1.2E-04	7	211	9.3
PI3K-Akt signaling pathway	1.7E-07	12	362	9.3
Pathways in cancer	1.0E-06	13	529	6.9
Metabolic pathways	1.6E-16	34	1556	6.1

Supplementary Table S8: Metabolite enrichment analysis of significantly dysregulated genes in MCF-7 cells following paclitaxel treatment, performed using the Metabolomics Workbench Metabolites 2022 database in Enrichr. Genes showing ≥2-fold upregulation or ≤0.5-fold downregulation were included in the analysis to identify metabolites associated with altered gene expression profiles. The table presents metabolite index numbers (Index), metabolite names (Name), raw p-values (p-value), adjusted p-values (Adjusted p-value), odds ratio values (Odds ratio), and combined enrichment scores (Combined score). Adjusted p-values were calculated to control for multiple comparisons, and statistical significance was considered at adjusted $p < 0.05$. Accessed May 16, 2026.

Index	Name	p-value	Adjusted p-value	Odds ratio	Combined score
1	Coenzyme A	9,03E-05	0.000006771	16.57	268.84
2	ATP	0.000001951	0.00007318	8.97	117.95
3	3-Methyl Pyruvic Acid	0.00001439	0.0003598	90.37	1007.51
4	AMP	0.00003079	0.0004853	15.56	161.63

5	NAD+	0.00003235	0.0004853	10.99	113.57
6	Palmitoyl-CoA	0.0001593	0.001707	33.87	296.19
7	6R-Tetrahydrofolic Acid	0.0001593	0.001707	33.87	296.19
8	Pyruvic Acid	0.0002169	0.002033	30.Eki	253.97
9	3-Mercaptopyruvic Acid	0.0004676	0.003763	89.56	686.75
10	Palmitic Acid	0.0006523	0.003763	71.65	525.53
11	Dihydroxyacetone Phosphate	0.0006523	0.003763	71.65	525.53
12	Fumaric Acid	0.0006523	0.003763	71.65	525.53
13	Glycerol	0.0006523	0.003763	71.65	525.53
14	Acetyl-CoA	0.001008	0.005397	16.92	116.77
15	Acetoacetyl-CoA	0.001110	0.005550	51.17	348.14
16	CDP	0.002754	0.01148	29.84	175.91
17	UTP	0.002754	0.01148	29.84	175.91
18	ITP	0.002754	0.01148	29.84	175.91
19	IDP	0.003605	0.01423	25.58	143.88
20	CTP	0.004563	0.01711	22.38	120.61
21	ADP	0.007067	0.02524	5.58	27.62

22	Oxoglutaric Acid	0.01667	0.05437	10.84	44.38
23	GTP	0.01667	0.05437	10.84	44.38
24	NADP+	0.01861	0.05815	5.62	22.40
25	1-Acylglycerol	0.02794	0.06156	44.38	158.79
26	dIDP	0.02794	0.06156	44.38	158.79
27	dITP	0.02794	0.06156	44.38	158.79
28	dUMP	0.02794	0.06156	44.38	158.79
29	Isocitric Acid	0.03343	0.06156	35.50	120.65
30	Lauroyl-CoA	0.03343	0.06156	35.50	120.65
31	Octanoyl-CoA	0.03343	0.06156	35.50	120.65
32	3-Phosphoglyceric Acid	0.03343	0.06156	35.50	120.65
33	Decanoyl-CoA	0.03343	0.06156	35.50	120.65
34	Deoxyuridine	0.03343	0.06156	35.50	120.65
35	dATP	0.03343	0.06156	35.50	120.65
36	dGTP	0.03343	0.06156	35.50	120.65
37	dTDP	0.03343	0.06156	35.50	120.65
38	dTMP	0.03343	0.06156	35.50	120.65

39	dTTP	0.03343	0.06156	35.50	120.65
40	Methionine	0.03889	0.06156	29.58	96.06
41	Myristoyl-CoA	0.03889	0.06156	29.58	96.06
42	2-Phospho-D-glycerate	0.03889	0.06156	29.58	96.06
43	3-Oxohexanoyl-CoA	0.03889	0.06156	29.58	96.06
44	Triacylglycerol	0.03889	0.06156	29.58	96.06
45	Fructose 1,6-Bisphosphate	0.03889	0.06156	29.58	96.06
46	UDP	0.04394	0.06156	6.27	19.59
47	Phosphoribosyl Pyrophosphate	0.04432	0.06156	25.36	79.02
48	Ribose 5-Phosphate	0.04432	0.06156	25.36	79.02
49	Succinyl-CoA	0.04432	0.06156	25.36	79.02
50	Tetrahydrobiopterin	0.04432	0.06156	25.36	79.02
51	dUDP	0.04432	0.06156	25.36	79.02
52	sn-Glycero-3-phosphate	0.04432	0.06156	25.36	79.02
53	Glyceraldehyde 3-Phosphate	0.04432	0.06156	25.36	79.02
54	Hexanoyl-CoA	0.04432	0.06156	25.36	79.02
55	3',5' Cyclic AMP	0.04973	0.06660	22.19	66.59

56	dCDP	0.04973	0.06660	22.19	66.59
57	Thiamine Diphosphate	0.05510	0.06774	19.72	57.16
58	alpha-D-Glucose 6-Phosphate	0.05510	0.06774	19.72	57.16
59	beta-D-Glucose 6-Phosphate	0.05510	0.06774	19.72	57.16
60	dGDP	0.05510	0.06774	19.72	57.16
61	Glucose 6-Phosphate	0.05510	0.06774	19.72	57.16
62	3',5' Cyclic GMP	0.06575	0.07586	16.13	43.91
63	Propanoyl-CoA	0.06575	0.07586	16.13	43.91
64	dADP	0.06575	0.07586	16.13	43.91
65	IMP	0.06575	0.07586	16.13	43.91
66	Butanoyl-CoA	0.07103	0.07951	14.79	39.11
67	FAD	0.07103	0.07951	14.79	39.11
68	Fructose 6-Phosphate	0.08151	0.08990	12.67	31.77
69	Succinic Acid	0.09186	0.09985	11.9	26.47
70	Glucuronic Acid	0.1072	0.1132	9.34	20.85
71	Glycine	0.1072	0.1132	9.34	20.85
72	GDP	0.1272	0.1325	7.71	15.90

73	Glutamic Acid	0.2120	0.2178	4.32	Haz.70
74	S-Adenosylhomocysteine	0.2297	0.2328	3.94	May.79
75	S-Adenosylmethionine	0.2470	0.2470	3.61	5.Haz

Supplementary Table S9: Metabolite enrichment analysis of significantly dysregulated genes in SK-BR-3 cells following paclitaxel treatment, performed using the Metabolomics Workbench Metabolites 2022 database in Enrichr. Genes showing ≥2-fold upregulation or ≤0.5-fold downregulation were included in the analysis to identify metabolites associated with altered gene expression profiles. The table presents metabolite index numbers (Index), metabolite names (Name), raw p-values (p-value), adjusted p-values (Adjusted p-value), odds ratio values (Odds ratio), and combined enrichment scores (Combined score). Adjusted p-values were calculated to control for multiple comparisons, and statistical significance was considered at adjusted $p < 0.05$. Accessed May 16, 2026.

**Index**	**Name**	**p-value**	**Adjusted p-value**	**Odds ratio**	**Combined score**
1	ATP	9,67E-06	7,64E-04	12.1	221.63
2	Coenzyme A	5,89E-05	0.000002327	17.58	292.73
3	AMP	0.000001186	0.00003124	20.32	277.20
4	3-Methyl Pyruvic Acid	0.00001222	0.0002413	95.61	1081.62
5	Acetyl-CoA	0.00003568	0.0005637	24.88	254.81
6	Palmitoyl-CoA	0.0001355	0.001785	35.84	319.17
7	Pyruvic Acid	0.0001846	0.002083	31.85	273.84
8	Isocitric Acid	0.0004194	0.003302	94.71	736.52
9	3-Mercaptopyruvic Acid	0.0004194	0.003302	94.71	736.52

10	2-Phospho-D-glycerate	0.0005851	0.003302	75.76	563.96
11	Palmitic Acid	0.0005851	0.003302	75.76	563.96
12	Fumaric Acid	0.0005851	0.003302	75.76	563.96
13	Glucosamine 6-Phosphate	0.0005851	0.003302	75.76	563.96
14	Glycerol	0.0005851	0.003302	75.76	563.96
15	ADP	0.0007709	0.004060	7.51	53.83
16	Oxoglutaric Acid	0.0008603	0.004248	17.90	126.37
17	Acetoacetyl-CoA	0.0009961	0.004629	54.11	374.00
18	CDP	0.002474	0.009773	31.56	189.40
19	UTP	0.002474	0.009773	31.56	189.40
20	ITP	0.002474	0.009773	31.56	189.40
21	NAD+	0.002619	0.009854	7.46	44.36
22	Fructose 6-Phosphate	0.002845	0.01022	29.13	170.76
23	IDP	0.003240	0.01113	27.5	155.03
24	CTP	0.004102	0.01350	23.66	130.06
25	6R-Tetrahydrofolic Acid	0.004569	0.01444	22.27	120.00
26	GDP	0.007248	0.02202	17.20	84.77
27	GTP	0.01504	0.04400	10.46	48.11

28	NADP+	0.01611	0.04546	5.95	24.56
29	Glutamic Acid	0.02124	0.05688	9.45	36.41
30	1-Acylglycerol	0.02647	0.05688	46.91	170.36
31	3-Sulfino-alanine	0.02647	0.05688	46.91	170.36
32	dIDP	0.02647	0.05688	46.91	170.36
33	dITP	0.02647	0.05688	46.91	170.36
34	Lauroyl-CoA	0.03168	0.05688	37.52	129.54
35	Mannose	0.03168	0.05688	37.52	129.54
36	Mannose 6-Phosphate	0.03168	0.05688	37.52	129.54
37	Octanoyl-CoA	0.03168	0.05688	37.52	129.54
38	3-Phosphoglyceric Acid	0.03168	0.05688	37.52	129.54
39	Uridine	0.03168	0.05688	37.52	129.54
40	Decanoyl-CoA	0.03168	0.05688	37.52	129.54
41	dATP	0.03168	0.05688	37.52	129.54
42	dGTP	0.03168	0.05688	37.52	129.54
43	dTDP	0.03168	0.05688	37.52	129.54
44	dTTP	0.03168	0.05688	37.52	129.54
45	Methionine	0.03686	0.05710	31.27	103.21

46	Myristoyl-CoA	0.03686	0.05710	31.27	103.21
47	3-Oxohexanoyl-CoA	0.03686	0.05710	31.27	103.21
48	Triacylglycerol	0.03686	0.05710	31.27	103.21
49	beta-D-Fructose	0.03686	0.05710	31.27	103.21
50	Fructose	0.03686	0.05710	31.27	103.21
51	Fructose 1,6-Bisphosphate	0.03686	0.05710	31.27	103.21
52	UDP	0.03982	0.05723	6.63	21.37
53	Phenylalanine	0.04201	0.05723	26.80	84.95
54	Phosphoribosyl Pyrophosphate	0.04201	0.05723	26.80	84.95
55	Ribose 5-Phosphate	0.04201	0.05723	26.80	84.95
56	Tetrahydrobiopterin	0.04201	0.05723	26.80	84.95
57	dUDP	0.04201	0.05723	26.80	84.95
58	Hexanoyl-CoA	0.04201	0.05723	26.80	84.95
59	Tyrosine	0.04714	0.06207	23.45	71.63
60	dCDP	0.04714	0.06207	23.45	71.63
61	Oxaloacetic Acid	0.05224	0.06253	20.84	61.53
62	UMP	0.05224	0.06253	20.84	61.53
63	alpha-D-Glucose 6-Phosphate	0.05224	0.06253	20.84	61.53

64	beta-D-Glucose 6-Phosphate	0.05224	0.06253	20.84	61.53
65	dGDP	0.05224	0.06253	20.84	61.53
66	Glucose 6-Phosphate	0.05224	0.06253	20.84	61.53
67	Serine	0.05731	0.06659	18.76	53.63
68	Cysteine	0.05731	0.06659	18.76	53.63
69	Propanoyl-CoA	0.06236	0.06842	17.5	47.31
70	Aspartic Acid	0.06236	0.06842	17.5	47.31
71	dADP	0.06236	0.06842	17.5	47.31
72	IMP	0.06236	0.06842	17.5	47.31
73	Butanoyl-CoA	0.06738	0.07292	15.63	42.16
74	CMP	0.07237	0.07726	14.43	37.88
75	Succinic Acid	0.08719	0.09184	11.72	28.59
76	Glucuronic Acid	0.1018	0.1044	9.87	22.55
77	Glycine	0.1018	0.1044	9.87	22.55
78	Glucose	0.1114	0.1128	8.93	19.59
79	S-Adenosylmethionine	0.2355	0.2355	3.82	5.52

Supplementary Table S10: Metabolite enrichment analysis of significantly dysregulated genes in BT-474 cells following paclitaxel treatment, performed using the Metabolomics Workbench Metabolites 2022 database in Enrichr. Genes showing ≥2-fold upregulation or ≤0.5-fold downregulation were included in the analysis to identify metabolites associated with altered gene expression profiles. The table presents

metabolite index numbers (Index), metabolite names (Name), raw p-values (p-value), adjusted p-values (Adjusted p-value), odds ratio values (Odds ratio), and combined enrichment scores (Combined score). Adjusted p-values were calculated to control for multiple comparisons, and statistical significance was considered at adjusted $p < 0.05$. Accessed May 16, 2026.

**Index**	**Name**	**p-value**	**Adjusted p-value**	**Odds ratio**	**Combined score**
1	ATP	9,10E-07	7,74E-05	13.4	271.36
2	Coenzyme A	3,49E-06	1,48E-04	19.80	385.59
3	AMP	5,65E-05	0.000001602	23.88	398.52
4	Palmitoyl-CoA	0.000003037	0.00006454	50.48	641.31
5	6R-Tetrahydrofolic Acid	0.0001432	0.002434	35.16	311.18
6	NAD+	0.0002998	0.004247	9.33	75.67
7	Methionine	0.0006071	0.005160	74.34	550.62
8	Palmitic Acid	0.0006071	0.005160	74.34	550.62
9	Dihydroxyacetone Phosphate	0.0006071	0.005160	74.34	550.62
10	Fumaric Acid	0.0006071	0.005160	74.34	550.62
11	Acetyl-CoA	0.0009077	0.006430	17.56	123.03
12	GTP	0.0009077	0.006430	17.56	123.03
13	Acetoacetyl-CoA	0.001033	0.006757	53.09	365.02
14	CDP	0.002566	0.01363	30.96	184.71
15	UTP	0.002566	0.01363	30.96	184.71

16	ITP	0.002566	0.01363	30.96	184.71
17	IDP	0.003360	0.01680	26.54	151.16
18	CTP	0.004253	0.02009	23.22	126.77
19	Pyruvic Acid	0.005776	0.02584	19.55	100.76
20	ADP	0.006232	0.02649	5.79	29.41
21	GDP	0.007511	0.03040	16.88	82.57
22	Oxoglutaric Acid	0.01558	0.06018	11.25	46.81
23	NADP+	0.01692	0.06254	5.84	23.80
24	1-Acylglycerol	0.02696	0.06270	46.03	166.35
25	3-Sulfino-alanine	0.02696	0.06270	46.03	166.35
26	dIDP	0.02696	0.06270	46.03	166.35
27	dITP	0.02696	0.06270	46.03	166.35
28	dUMP	0.02696	0.06270	46.03	166.35
29	S-Adenosylmethionine	0.03042	0.06270	8.73	26.99
30	Isocitric Acid	0.03226	0.06270	36.83	126.46
31	Lauroyl-CoA	0.03226	0.06270	36.83	126.46
32	Octanoyl-CoA	0.03226	0.06270	36.83	126.46
33	3-Mercaptopyruvic Acid	0.03226	0.06270	36.83	126.46

34	3-Phosphoglyceric Acid	0.03226	0.06270	36.83	126.46
35	Uridine	0.03226	0.06270	36.83	126.46
36	Decanoyl-CoA	0.03226	0.06270	36.83	126.46
37	Deoxyuridine	0.03226	0.06270	36.83	126.46
38	dATP	0.03226	0.06270	36.83	126.46
39	dGTP	0.03226	0.06270	36.83	126.46
40	dTDP	0.03226	0.06270	36.83	126.46
41	dTMP	0.03226	0.06270	36.83	126.46
42	dTTP	0.03226	0.06270	36.83	126.46
43	Myristoyl-CoA	0.03754	0.06270	30.69	100.73
44	2-Phospho-D-glycerate	0.03754	0.06270	30.69	100.73
45	3-Oxohexanoyl-CoA	0.03754	0.06270	30.69	100.73
46	Fructose 1,6-Bisphosphate	0.03754	0.06270	30.69	100.73
47	Glucosamine 6-Phosphate	0.03754	0.06270	30.69	100.73
48	Glycerol	0.03754	0.06270	30.69	100.73
49	UDP	0.04118	0.06270	6.50	20.75
50	Phenylalanine	0.04278	0.06270	26.30	82.89
51	Phosphoribosyl Pyrophosphate	0.04278	0.06270	26.30	82.89

52	Ribose 5-Phosphate	0.04278	0.06270	26.30	82.89
53	Succinyl-CoA	0.04278	0.06270	26.30	82.89
54	Tetrahydrobiopterin	0.04278	0.06270	26.30	82.89
55	dUDP	0.04278	0.06270	26.30	82.89
56	sn-Glycero-3-phosphate	0.04278	0.06270	26.30	82.89
57	Glyceraldehyde 3-Phosphate	0.04278	0.06270	26.30	82.89
58	Hexanoyl-CoA	0.04278	0.06270	26.30	82.89
59	3',5' Cyclic AMP	0.04800	0.06553	23.1	69.88
60	3-Methyl Pyruvic Acid	0.04800	0.06553	23.1	69.88
61	Tyrosine	0.04800	0.06553	23.1	69.88
62	dCDP	0.04800	0.06553	23.1	69.88
63	Oxaloacetic Acid	0.05319	0.06553	20.45	60.01
64	Thiamine Diphosphate	0.05319	0.06553	20.45	60.01
65	UMP	0.05319	0.06553	20.45	60.01
66	alpha-D-Glucose 6-Phosphate	0.05319	0.06553	20.45	60.01
67	beta-D-Glucose 6-Phosphate	0.05319	0.06553	20.45	60.01
68	dGDP	0.05319	0.06553	20.45	60.01
69	Glucose 6-Phosphate	0.05319	0.06553	20.45	60.01

70	Serine	0.05836	0.06986	18.41	52.30
71	Cysteine	0.05836	0.06986	18.41	52.30
72	3',5' Cyclic GMP	0.06349	0.07101	16.73	46.13
73	Propanoyl-CoA	0.06349	0.07101	16.73	46.13
74	Aspartic Acid	0.06349	0.07101	16.73	46.13
75	dADP	0.06349	0.07101	16.73	46.13
76	IMP	0.06349	0.07101	16.73	46.13
77	Butanoyl-CoA	0.06860	0.07475	15.34	41.10
78	FAD	0.06860	0.07475	15.34	41.10
79	CMP	0.07368	0.07927	14.16	36.93
80	Fructose 6-Phosphate	0.07873	0.08365	13.15	33.41
81	Succinic Acid	0.08875	0.09313	11.50	27.86
82	Glucuronic Acid	0.1036	0.1061	9.68	21.96
83	Glycine	0.1036	0.1061	9.68	21.96
84	Glutamic Acid	0.2053	0.2077	4.48	7.10
85	S-Adenosylhomocysteine	0.2225	0.2225	4.9	6.14

Supplementary Table S11: Metabolite enrichment analysis of significantly dysregulated genes in MDA-MB-231 cells following paclitaxel treatment, performed using the Metabolomics Workbench Metabolites 2022 database in Enrichr. Genes showing ≥2-fold upregulation or ≤0.5-fold downregulation were included in the analysis to identify metabolites associated with altered gene expression profiles. The table presents

metabolite index numbers (Index), metabolite names (Name), raw p-values (p-value), adjusted p-values (Adjusted p-value), odds ratio values (Odds ratio), and combined enrichment scores (Combined score). Adjusted p-values were calculated to control for multiple comparisons, and statistical significance was considered at adjusted $p < 0.05$. Accessed May 16, 2026.

**Index**	**Name**	**p-value**	**Adjusted p-value**	**Odds ratio**	**Combined score**
1	ATP	2,09E-04	0.000009685	11.98	184.20
2	Coenzyme A	2,48E-04	0.000009685	18.85	286.70
3	AMP	0.000008639	0.0002246	20.52	239.23
4	Oxoglutaric Acid	0.00001578	0.0003076	30.91	341.76
5	Palmitoyl-CoA	0.00007325	0.0009523	44.41	422.88
6	6R-Tetrahydrofolic Acid	0.00007325	0.0009523	44.41	422.88
7	NAD+	0.0001040	0.001159	11.84	108.61
8	Methionine	0.0003873	0.002746	93.68	736.02
9	Palmitic Acid	0.0003873	0.002746	93.68	736.02
10	Dihydroxyacetone Phosphate	0.0003873	0.002746	93.68	736.02
11	Glycerol	0.0003873	0.002746	93.68	736.02
12	GTP	0.0004705	0.003058	22.19	170.00
13	3-Methyl Pyruvic Acid	0.0006602	0.003678	66.91	489.99
14	Acetoacetyl-CoA	0.0006602	0.003678	66.91	489.99
15	NADP+	0.0009180	0.004773	10.5	70.31

16	Pyruvic Acid	0.003723	0.01815	24.64	137.80
17	GDP	0.004851	0.02226	21.27	113.36
18	Acetyl-CoA	0.01014	0.04394	14.17	65.08
19	Glutamic Acid	0.01439	0.05571	11.69	49.58
20	1-Acylglycerol	0.02156	0.05571	57.88	222.05
21	3-Sulfino-alanine	0.02156	0.05571	57.88	222.05
22	dIDP	0.02156	0.05571	57.88	222.05
23	dITP	0.02156	0.05571	57.88	222.05
24	Isocitric Acid	0.02582	0.05571	46.30	169.29
25	Lauroyl-CoA	0.02582	0.05571	46.30	169.29
26	Octanoyl-CoA	0.02582	0.05571	46.30	169.29
27	3-Mercaptopyruvic Acid	0.02582	0.05571	46.30	169.29
28	3-Phosphoglyceric Acid	0.02582	0.05571	46.30	169.29
29	Uridine	0.02582	0.05571	46.30	169.29
30	Decanoyl-CoA	0.02582	0.05571	46.30	169.29
31	dATP	0.02582	0.05571	46.30	169.29
32	dGTP	0.02582	0.05571	46.30	169.29
33	dTDP	0.02582	0.05571	46.30	169.29

34	dTTP	0.02582	0.05571	46.30	169.29
35	Myristoyl-CoA	0.03006	0.05571	38.58	135.20
36	2-Phospho-D-glycerate	0.03006	0.05571	38.58	135.20
37	3-Oxohexanoyl-CoA	0.03006	0.05571	38.58	135.20
38	Triacylglycerol	0.03006	0.05571	38.58	135.20
39	Fumaric Acid	0.03006	0.05571	38.58	135.20
40	Phenylalanine	0.03428	0.05571	33.07	111.54
41	Phosphoribosyl Pyrophosphate	0.03428	0.05571	33.07	111.54
42	Ribose 5-Phosphate	0.03428	0.05571	33.07	111.54
43	Succinyl-CoA	0.03428	0.05571	33.07	111.54
44	Tetrahydrobiopterin	0.03428	0.05571	33.07	111.54
45	dUDP	0.03428	0.05571	33.07	111.54
46	sn-Glycero-3-phosphate	0.03428	0.05571	33.07	111.54
47	Glyceraldehyde 3-Phosphate	0.03428	0.05571	33.07	111.54
48	Hexanoyl-CoA	0.03428	0.05571	33.07	111.54
49	3',5' Cyclic AMP	0.03848	0.05738	28.93	94.25
50	Tyrosine	0.03848	0.05738	28.93	94.25
51	dCDP	0.03848	0.05738	28.93	94.25

52	Oxaloacetic Acid	0.04267	0.05738	25.72	81.12
53	Thiamine Diphosphate	0.04267	0.05738	25.72	81.12
54	UMP	0.04267	0.05738	25.72	81.12
55	alpha-D-Glucose 6-Phosphate	0.04267	0.05738	25.72	81.12
56	beta-D-Glucose 6-Phosphate	0.04267	0.05738	25.72	81.12
57	dGDP	0.04267	0.05738	25.72	81.12
58	Glucose 6-Phosphate	0.04267	0.05738	25.72	81.12
59	Serine	0.04683	0.06088	23.14	70.84
60	Cysteine	0.04683	0.06088	23.14	70.84
61	3',5' Cyclic GMP	0.05098	0.06213	21.4	62.61
62	Propanoyl-CoA	0.05098	0.06213	21.4	62.61
63	Aspartic Acid	0.05098	0.06213	21.4	62.61
64	dADP	0.05098	0.06213	21.4	62.61
65	Butanoyl-CoA	0.05511	0.06513	19.28	55.89
66	FAD	0.05511	0.06513	19.28	55.89
67	CDP	0.05923	0.06599	17.80	50.31
68	CMP	0.05923	0.06599	17.80	50.31
69	UTP	0.05923	0.06599	17.80	50.31

70	ITP	0.05923	0.06599	17.80	50.31
71	IDP	0.06740	0.07404	15.42	41.60
72	Succinic Acid	0.07146	0.07741	14.46	38.15
73	CTP	0.07550	0.08067	13.61	35.16
74	Glucuronic Acid	0.08353	0.08688	12.18	30.22
75	Glycine	0.08353	0.08688	12.18	30.22
76	ADP	0.1155	0.1186	3.53	7.61
77	S-Adenosylmethionine	0.1961	0.1986	4.71	7.68
78	UDP	0.2271	0.2271	3.98	5.90

Supplementary Table S12 : RT-qPCR-based analysis of gene expression for drug resistance-related primers listed in Supplementary Table 2 in MCF-7, SK-BR-3, BT-474, and MDA-MB-231 cell lines. Relative expression levels were calculated using the 2^−ΔΔCt method and normalized to the control group (set as 1). All experiments were performed in triplicate. Statistical analysis was conducted using two-way ANOVA with comparisons made against the control group, followed by Dunnett's multiple comparisons test. Data are presented as mean values, and statistical significance is indicated as follows: *p < 0.05, **p < 0.01, ***p < 0.001, and **p < 0.0001.

Gene	MCF-7	SK-BR-3	BT-474	MDA-MB-231	Gene	MCF-7	SK-BR-3	BT474	MDA-MB-231
ABCB1	3.33 ± 0.95	1.99 ± 0.59	9.12 ± 0.73*	2.43 ± 0.33	ERCC3	0.23 ± 1.32	-6.26 ± 1.31	-3.28 ± 1.22	1.80 ± 0.74
ABCC1	10.01 ± 2.47***	1.55 ± 0.22	2.64 ± 1.02	-1.89 ± 0.13	FOS	6.21 ± 1.63	-2.63 ± 067	-3.93 ± 1.69	5.09 ± 2.21
ABCC2	3.32 ± 0.92	3.17 ± 1.77	6.11 ± 1.05	0.51 ± 1.70	GSK3A	1.72 ± 0.44	-1.96 ± 3.21	1.73 ± 0.58	3.58 ± 1.47
ABCC3	19.50 ± 5.13***	1.47 ± 0.10	-0.76 ± 1.77	2.38 ± 1.08	HIF1A	3.08 ± 0.71	-1.38 ± 2.62	2.49 ± 0.80	-1.96 ± 0.73
ABCC5	6.04 ± 1.59	1.76 ± 0.42	9.81 ± 0.75**	4.08 ± 1.84	MSH2	-3.48 ± 0.89	2.86 ± 1.58	0.14 ± 1.45	-2.36 ± 092
ABCG2	5.92 ± 1.50	1.75 ± 0.38	9.57 ± 0.63**	2.67 ± 1.15	MVP	14.23 ± 2.75**	1.94 ± 0.55	17.85 ± 1.05****	-1.64 ± 0.35

APC	0.26 ± 1.33	-15.51 ± 2.74****	0.46 ± 1.56	2.07 ± 0.85	RB1	5.43 ± 1.42	1.24 ± 0.08	4.13 ±0.83	1.85 ± 0.74
ATM	-1.78 ± 0.18	-2.13 ± 3.41	2.40 ±0.85	0.04 ± 1.56	SOD1	0.01 ± 1.26	-13.99 ± 1.52****	-0.15 ± 1.33	-1.63 ± 0.54
BCL2	0.27 ± 1.37	-2.20 ± 0.07	0.26 ± 1.60	0.12 ± 1.66	SULT1E1	-3.17 ± 0.92	0.08 ± 1.26	-0.35 ± 1.57	2.33 ± 1.05
BCL2L1	5.53 ± 1.43	2.17 ± 0.84	4.12 ± 0.51	1.89 ± 0.74	TOP1	4.30 ±1.04	1.34 ± 0.05	-0.11 ± 1.50	0.16 ± 1.49
BLMH	-1.47 ± 0.39	-3.06 ± 4.35	-0.12 ± 1.48	0.16 ± 1.58	TOP2A	-2.24 ± 0.58	1.29 ± 0.03	-5.81 ± 2.36	0.18 ± 1.46
BOX	7.47 ±1.91*	1.64 ± 0.30	14.99 ± 0.47****	2.23 ± 0.92	TOP2B	3.66 ± 0.88	0.12 ± 1.14	-0.07 ± 1.44	0.26 ± 1.48
BRCA1	-10.89 ± 3.08*	-14.66 ± 0.69****	1.74 ±0.63	0.14 ± 1.58	TPMT	1.69 ± 0.42	-2.50 ± 0.42	-0.08 ± 1.42	2.80 ± 1.12
BRCA2	-1.77 ± 0.48	1.65 ± 0.34	4.92 ±0.81	0.42 ± 1.67	UGCG	1.35 ± 0.34	-4.69 ± 1.66*	0.45 ± 1.54	0.54 ± 1.59
CLPTM1L	-4.12 ± 1.10	-10.99 ± 0.29***	0.38 ± 1.47	2.51 ± 1.05	XPA	1.93 ±0.48	-12.00 ± 1.22****	1.67 ± 0.60	0.39 ± 1.56
CMYC	2.22 ± 0.51	-3.54 ± 4.74	0.01 ± 1.37	-2.48 ± 0.92	XPC	7.71 ± 2.27**	1.47 ± 0.07	4.25 ± 1.18	3.35 ± 1.60
EPHX1	2.61 ± 0.67	-14.16 ± 0.19****	2.63 ± 0.89	0.28 ± 1.62					
